# A REDD1/TXNIP pro-oxidant complex regulates ATG4B activity to control stress-induced autophagy and sustain exercise capacity

**DOI:** 10.1038/ncomms8014

**Published:** 2015-04-28

**Authors:** Shuxi Qiao, Michael Dennis, Xiufeng Song, Douangsone D. Vadysirisack, Devika Salunke, Zachary Nash, Zhifen Yang, Marc Liesa, Jun Yoshioka, Shu-Ichi Matsuzawa, Orian S. Shirihai, Richard T. Lee, John C. Reed, Leif W. Ellisen

**Affiliations:** 1 Massachusetts General Hospital Cancer Center, Boston, Massachusetts 02114, USA; 2 Harvard Medical School, Boston, Massachusetts 02115, USA; 3 Sanford-Burnham Medical Research Institute, La Jolla, California 92037, USA; 4 Department of Medicine, Evans Center, Mitochondria ARC, Boston University School of Medicine, Boston, Massachusetts 02118, USA; 5 Regenerative Medicine Center, Brigham and Women's Hospital, Boston, Massachusetts 02115, USA; 6 Department of Clinical Biochemistry, Faculty of Medicine, Ben Gurion University of the Negev, Beer-Sheva 8410501, Israel; 7 Roche Pharmaceutical Research and Early Development, Basel 4070, Switzerland

## Abstract

Macroautophagy (autophagy) is a critical cellular stress response; however, the signal transduction pathways controlling autophagy induction in response to stress are poorly understood. Here we reveal a new mechanism of autophagy control whose deregulation disrupts mitochondrial integrity and energy homeostasis *in vivo*. Stress conditions including hypoxia and exercise induce reactive oxygen species (ROS) through upregulation of a protein complex involving REDD1, an mTORC1 inhibitor and the pro-oxidant protein TXNIP. Decreased ROS in cells and tissues lacking either REDD1 or TXNIP increases catalytic activity of the redox-sensitive ATG4B cysteine endopeptidase, leading to enhanced LC3B delipidation and failed autophagy. Conversely, REDD1/TXNIP complex expression is sufficient to induce ROS, suppress ATG4B activity and activate autophagy. In *Redd1*^*−/−*^ mice, deregulated ATG4B activity and disabled autophagic flux cause accumulation of defective mitochondria, leading to impaired oxidative phosphorylation, muscle ATP depletion and poor exercise capacity. Thus, ROS regulation through REDD1/TXNIP is physiological rheostat controlling stress-induced autophagy.

## Abstract (web summary)

Stress-induced macroautophagy is initiated by the induction of reactive oxygen species (ROS). Here Qiao *et al.* show that the mTOR inhibitor REDD1 in a complex with pro-oxidant protein TXNIP induces ROS formation, leading to ATG4B suppression and autophagy activation in a largely mTOR-independent manner.

## Graphical abstract

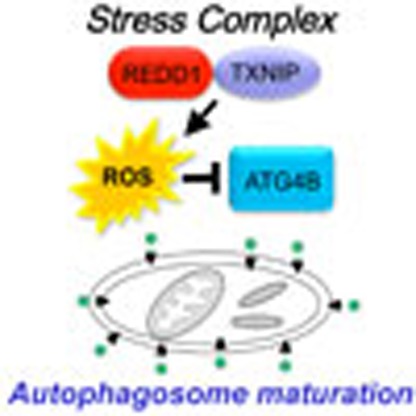

Macroautophagy (hereafter autophagy) is an evolutionarily conserved process of self-catabolism^1^,^2^. While basal autophagy serves to degrade aged and defective cellular organelles and macromolecules for reprocessing, autophagy is also activated under conditions of hypoxia and nutrient depletion as a means to provide bioenergetic intermediates^3^. The induction of autophagy involves the assembly of a double-membrane autophagosome that subsequently delivers its cargo to lysosomes for degradation. Regulation of this process involves a limited set of highly conserved autophagy genes (ATGs) initially identified in yeast^4^. Although the assembly of the autophagosome in response to metabolic stress is known to be regulated at multiple steps, a detailed understanding of the mechanisms involved is lacking.

A key regulator of autophagy in mammalian cells is the mTOR kinase complex 1 (mTORC1). This kinase complex monitors nutrient and growth factor availability, receiving multiple upstream signals, which it integrates to control diverse biological processes including protein translation and bioenergetic organization^5^. Under nutrient replete conditions, activated mTORC1 leads to suppression of autophagy through phosphorylation of proximal components of the autophagy machinery including the Unk-51-like kinase ULK1 and Atg13 (refs 6, 7). In response to hypoxia, nutrient deprivation or energy stress, mTORC1 is inhibited, leading to activation of autophagy and self-catabolism required to restore homeostasis^8^. The importance of coordinating mTORC1 activation with autophagy suppression is underscored by the observation that the key regulators of mTORC1 control autophagy at multiple levels. For example, the AMP-activated kinase AMPK, a central sensor of energy stress, inhibits mTORC1 activity and activates autophagy at multiple levels through phosphorylation of distinct substrates. These include the tuberous sclerosis complex protein TSC2, an upstream mTORC1 inhibitor; raptor, an intrinsic mTORC1 component; ULK1, a key autophagy initiator; and VPS34, a class III phosphatidylinositol-3-kinase involved in autophagy and membrane trafficking^7^,^9^.

Among the essential regulators of mTORC1 activity in response to hypoxia and energy stress is REDD1 (DDIT4, Dig2 and RTP801). REDD1 and its conserved orthologues in *Drosophila*, Scylla and Charybdis are induced under stress conditions and rapidly inactivate mTORC1 in a TSC1/2-dependent manner^10^,^11^,^12^,^13^. REDD1 functions as a molecular shuttle to remove inhibitory 14-3-3 proteins from TSC2, and REDD1 is both necessary and sufficient for this biochemical effect^14^. Genetic deletion of REDD1 protects certain tissues from particular stress stimuli, including oxidative retinopathy of prematurity and cigarette smoke (CS)-induced lung damage^15^,^16^. In this regard, it is notable that REDD1 has also been associated in multiple studies with induction of cellular reactive oxygen species (ROS)^17^,^18^. In addition, a tumour-suppressive effect of REDD1 is supported by its p53-dependent regulation and its transcriptional silencing in a subset of human cancers^14^,^17^,^19^. Notably, however, REDD1-dependent ROS regulation, stress protection phenotypes and tumour suppression are all at least in part mTORC1-independent, arguing for an unidentified REDD1-dependent pathway that mediates these effects^16^,^20^.

Here we use biochemical approaches and *in vivo* models to uncover a new pathway for basal and stress-induced autophagy, requiring REDD1 but largely independent of mTORC1. This finding is explained by our discovery that REDD1 forms a stress-induced complex with the pro-oxidant protein TXNIP (VDUP1 and TBP2) to induce ROS. In the absence of REDD1, decreased cytosolic ROS results in hyperactivation of the cysteine endopeptidase ATG4B, resulting in delipidation of the microtubule-associated protein 1 light chain 3 (LC3) and defective autophagosome assembly. Failed mitochondrial clearance due to impaired autophagy in this setting results in defective oxidative phosphorylation, ATP depletion and poor exercise capacity of *Redd1*^*−/−*^ mice. Collectively, these findings reveal the detailed mechanism linking ROS regulation to stress-induced autophagy, energy homeostasis and exercise physiology.

## Results

### Defective autophagy in *Redd1* ^−/−^ tissues and cells

**Figure 1:**
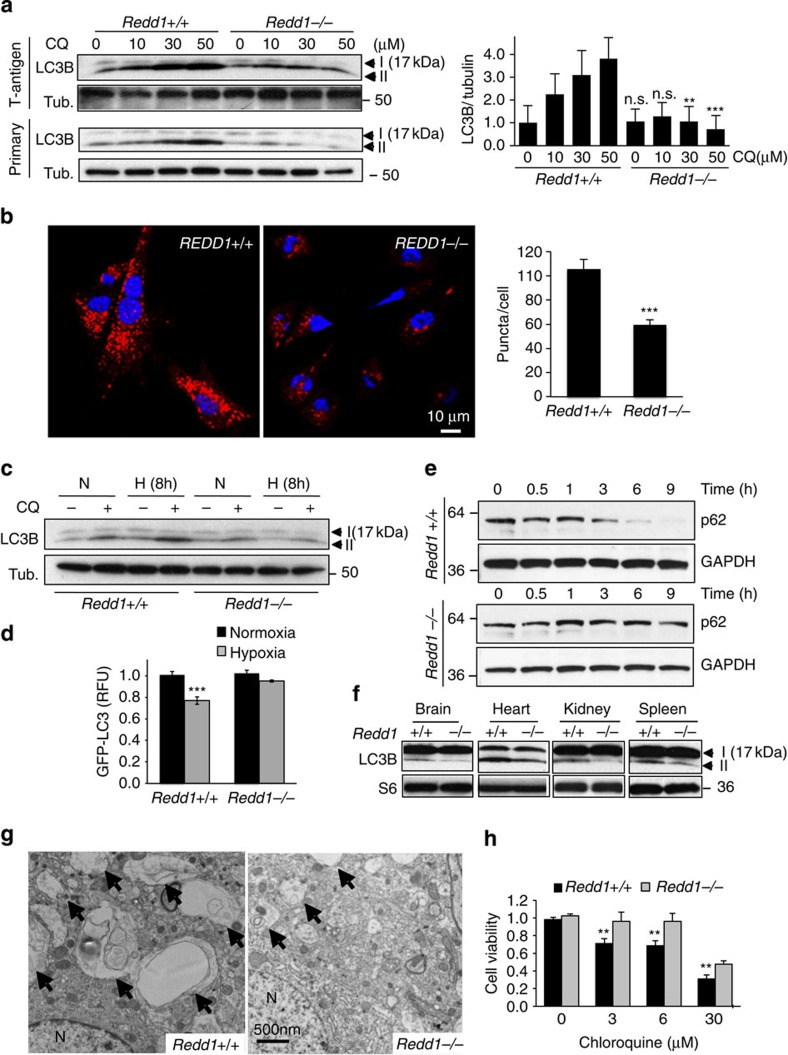
Loss of REDD1 results in defective autophagy *in vitro* and *in vivo*. (**a**). Western blot analysis showing reduced lipidated LC3B (LC3B-II, bottom band) in immortalized (top) and primary (bottom) *Redd1*^*−/−*^ cells under basal conditions. Autophagic flux was assessed using CQ at the indicated concentrations for 4 h. β-Tubulin (Tub.) serves as a loading control. At right, densitometry showing the mean values of three independent experiments and statistical comparison with respective wild-type lanes. (**b**) Representative confocal immunofluorescence images showing decreased LC3B membrane-associated puncta in *Redd1*^*−/−*^ compared with wild-type cells cultured under basal conditions in the presence of CQ (30 μM, 4 h). *N*≥60 cells per genotype were analysed as described in Methods. At right, quantitation of three independent experiments. (**c**) Impaired hypoxia-induced autophagy in *Redd1*^*−/−*^ cells. Cells were cultured under normoxia (N) or hypoxia (H, 1% O_2_) and treated in the absence or presence of CQ (30 μM, 4 h). (**d**) Attenuated hypoxia-induced degradation of GFP-LC3 in *Redd1*^*−/−*^ cells. Retrovirally transduced cells were cultured under normoxia or hypoxia (1% O_2_, 4 h), and analysed using flow cytometry. (**e**) Failed p62 degradation under starvation (Krebs medium) in *Redd1*^*−/−*^ cells. (**f**) Disabled autophagy in tissues from *Redd1*^*−/−*^ mice, evidenced by reduced processing of LC3B post 24-h starvation. Ribosomal protein S6, loading control. (**g**) Electron microscopy showing decreased number and size of autophagic bodies (arrows) in the brain of *Redd1*^*−/−*^ mice following starvation for 24 h. N indicates nuclei. (**h**) Reduced sensitivity of *Redd1*^*−/−*^ cells to autophagy inhibition, determined with crystal violet staining at 72 h. For **d**,**h**, shown are the means of two independent experiments performed as triplicate cultures. All error bars indicate s.d. ***P<0.001, **P<0.01, n.s. not significant, by paired *t*-test (**d**,**h**) or unpaired *t*-test (**b**).

In order to understand the contribution of REDD1 to metabolic homeostasis, we generated mice and cells with constitutive deletion of the entire REDD1-coding region^12^. We first examined basal and stress-induced autophagy in primary and immortalized wild-type and matched *Redd1*^*−/−*^ mouse embryo fibroblasts (MEFs). The microtubule-associated protein 1 LC3 (the yeast Atg8 homologue) is a key factor required for elongation and eventual closure of the autophagosome^21^. LC3 is processed by cleavage (producing LC3-I) and subsequent lipid conjugation (yielding LC3-II) for membrane targeting, and thus LC3-II induction is an established marker of autophagy^22^. We observed substantially lower levels of LC3-II in *Redd1*^*−/−*^ cells despite no difference in total LC3 expression (Fig. 1a and Supplementary Fig. 1a). This difference was particularly apparent when autophagy was blocked by chloroquine (CQ), an inhibitor of lysosomal acidification and autophagosome degradation (Fig. 1a)^22^. Defective autophagy was corroborated using confocal immunofluorescence analysis of endogenous LC3, which showed decreased LC3 membrane-associated puncta in *Redd1*^*−/−*^ cells (Fig. 1b). Similarly, decreased LC3 puncta in the absence of REDD1 was also observed following transfection of green fluorescent protein (GFP)-tagged LC3 (Supplementary Fig. 1b).

Strikingly, the physiologic induction of autophagy in response to hypoxic stress was also severely impaired in the absence of REDD1 (Fig. 1c and Supplementary Fig. 1c,d). We further interrogated the autophagic flux in this setting by measuring degradation of GFP-tagged LC3, a sensitive and quantitative indicator of autophagy, which was indeed highly attenuated in response to hypoxia in *Redd1*^*−/−*^ cells (Fig. 1d)^23^. Degradation of p62, a key autophagy substrate^24^, was remarkably impaired in *Redd1*^*−/−*^ cells in response to starvation, further supporting a requirement for REDD1 in stress-induced autophagy (Fig. 1e). *In vivo*, brains of starved *Redd1*^*−/−*^ mice demonstrated a dramatic reduction in both the size and number of autophagic vesicles assessed using electron microscopy (Fig. 1g), which correlated with defective LC3 lipidation in this and several other tissues (Fig. 1g). Finally, since autophagy is important for metabolic homeostasis in many actively cycling cells, we examined the effect of autophagy inhibition on cell proliferation. We found that CQ strongly reduced proliferation of wild-type cells, while *Redd1*^*−/−*^ cells, which have adapted to survive with defective autophagy, were less sensitive to the CQ treatment (Fig. 1h). Thus, basal and stress-induced autophagy are impaired in the absence of REDD1, both *in vitro* and *in vivo*.

### REDD1 regulates autophagy independent of mTORC1

**Figure 2:**
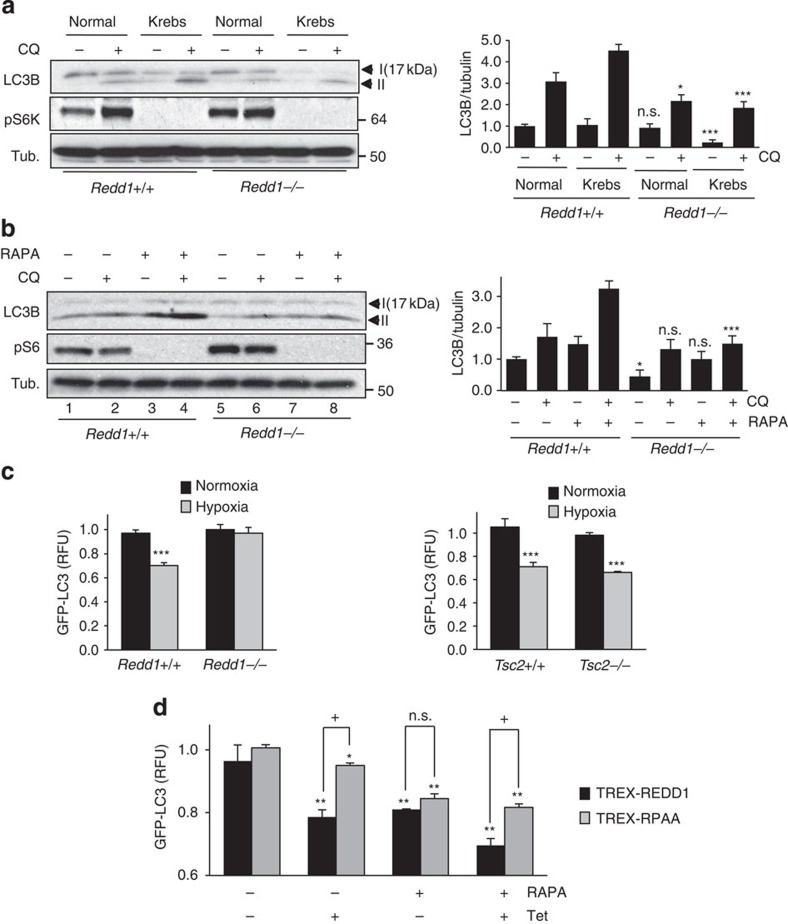
REDD1 mediates autophagy independent of mTORC1. (**a**) Impaired starvation-induced autophagy in *Redd1*^*−/−*^ cells despite suppressed mTORC1, assessed by the analysis of phosphorylated p70 S6 Kinase (pS6K, T389). Cells were cultured with Krebs media and treated in the absence or presence of CQ (30 μM, 1 h). At right, densitometry showing the mean values of three independent experiments and statistical comparison with respective wild-type lanes. (**b**) Rapamycin (100 nM, 4 h) fails to fully restore the defective autophagic flux in *Redd1*^*−/−*^ cells, as assessed by LC3B processing in the absence or presence of CQ (30 μM, 4 h; compare lanes 4 and 8). Activity of mTORC1 was assessed via phosphorylated ribosomal protein S6 (pS6, S235/S236). Right, densitometry quantitation of three independent experiments as in **a**. (**c**) Failure of hypoxia (1% O_2_, 12 h) to induce autophagy in *Redd1*^*−/−*^ cells (left) but not *Tsc2*^*−/−*^ cells (right), assessed by degradation of GFP–LC3 measured using flow cytometry. (**d**) Both wild-type REDD1 and the mTORC1-inactive mutant REDD1-RPAA^14^ are sufficient to induce autophagy, assessed by GFP-LC3 levels using flow cytometry. Tetracycline (Tet)-inducible (TREX) cells were treated in the presence and absence of rapamycin (100 nM, 4 h) as a positive control. For **c**,**d**, shown are the means of two independent experiments, each performed as triplicate cultures; error bars, s.d. (*) show *P*-values compared with untreated control; (**+**) compares REDD1 with REDD1-RPAA. For all experiments: ***P<0.001, **P<0.01, *P<0.05; ^+^*P*<0.05, by paired *t*-test.

As an established inhibitor of mTORC1, we wished to determine whether loss of REDD1 suppressed autophagy strictly through deregulation of mTORC1 activity. That this might not be the case was initially suggested by our observation that autophagy in response to starvation was defective in *Redd1*^*−/−*^ cells (Fig. 1), given that *Redd1* is not required for mTORC1 suppression in this setting^12^. Indeed, autophagy failed to be significantly induced following starvation in *Redd1*^*−/−*^ compared with wild-type cells, despite the potent suppression of mTORC1 we observed in cells of both genotypes (Fig. 2a). We next treated *Redd1*^*−/−*^ cells with either Torin2 or rapamycin, potent and specific mTORC1 suppressors^25^. While these agents induced autophagy as anticipated, they did not fully restore defective autophagy in *Redd1*^*−/−*^ cells (Fig. 2b and Supplementary Fig. 2a). Furthermore, cells deficient in TSC2, an mTORC1 inhibitor that is required for REDD1-mediated effects on mTORC1 (ref. 12), were competent for hypoxia-induced autophagy as recently reported^26^; however, *Redd1*^*−/−*^ cells were defective (Fig. 2c). Having shown that REDD1 is *necessary* for proper induction of stress-induced autophagy, we then asked whether REDD1 is *sufficient* to induce autophagy either dependent or independent of mTORC1 activity. We thus generated cells expressing wild-type REDD1 through a tetracycline-regulated promoter. Indeed, induction of REDD1 was sufficient to increase the autophagic flux as assessed by GFP-LC3 degradation (Fig. 2d and Supplementary Fig. 2b). Furthermore, induction of a REDD1 point mutant that does not inhibit mTORC1, REDD1-RPAA^14^, was also sufficient to induce LC3 degradation, although as expected this mutant was not as potent as wild-type REDD1 (Fig. 2d). Thus, REDD1 is both necessary and sufficient to induce autophagy through both mTORC1-dependent and mTORC1-independent mechanisms.

### REDD1 loss disables mitochondria and oxidative metabolism

**Figure 3:**
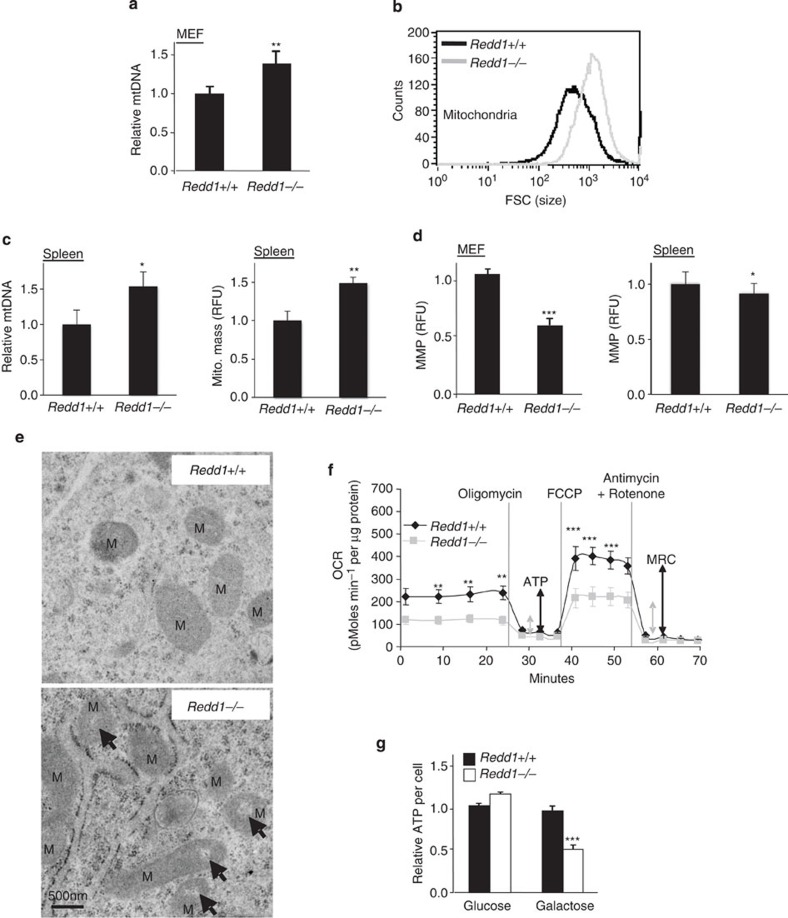
Loss of REDD1 results in accumulation of dysfunctional mitochondria. *Redd1*^*−/−*^ cells exhibit (**a**) increased mitochondrial (mt) number, evidenced by increased mitochondrial to nuclear (M/N) DNA ratio (COXII/β-globin) as measured by QRT–PCR. MEF, mouse embryonic fibroblast. PMEF, primary MEF. Shown are the mean from two independent experiments, each performed as triplicate cultures. (**b**) Increased mitochondrial size as determined using flow cytometry of cell-free, purified mitochondria. (**c**) Increased mitochondrial number (left) and mass (right) in *Redd1*^*−/−*^ primary splenocytes from starved mice (24 h), assessed, respectively, by M/N DNA ratio (*N*=4 mice per genotype, analysed in duplicate) and by staining with Mitotracker Green (100 nM) and then flow cytometry analysis (*N*=3 mice per genotype, analysed in triplicate). (**d**) Reduced mitochondrial membrane potential (MMP) in *Redd1*^*−/−*^ MEFs (left) and splenocytes (right) as assessed by staining with Tetramethylrhodamine, Ethyl Ester (TMRE, 100 nM) and then flow cytometry (*N*=2 mice per genotype, analysed in triplicate). (**e**) Dysmorphic mitochondria (M) in *Redd1*^*−/−*^ cells as assessed using electron microscopy. Arrows indicate abnormal hypodense regions. (**f**) Decreased basal O_2_ consumption rate (OCR), mitochondrial ATP synthesis (ATP) and maximal respiratory capacity (MRC) in *Redd1*^*−/−*^ cells, measured via Seahorse XF24. (**g**) Impaired oxidative phosphorylation in *Redd1*^*−/−*^ MEFs, shown by a drop in ATP per cell on shift from glucose to galactose-containing media (48 h). All error bars show s.d. ***P<0.001, **P<0.01, by paired *t*-test (**a**,**d**,**e**,**g**,**h**).

Multiple distinct genetic models of defective autophagy have revealed abnormalities in mitochondrial number and function as a prominent phenotype, resulting from a failure to eliminate aged, defective mitochondria^24^,^27^,^28^. Similarly, we observed an increase in both the number and size of mitochondria in the absence of REDD1, which was apparent not only in cultured cells but also in tissues of *Redd1*^*−/−*^ mice (Fig. 3a–c and Supplementary Fig. 3a). These mitochondria appeared functionally defective, as they are depolarized (Fig. 3d) and dysmorphic on electron microscopy analysis (Fig. 3e). Consistent with these defects, we previously reported that mitochondria from *Redd1*^*−/−*^ cells emit increased ROS^20^. Furthermore, we provide specific evidence for attenuated mitochondrial turnover in the absence of REDD1. We found that the mitochondrial protein TOM20 failed to be cleared on starvation in *Redd1*^*−/−*^ cells (Supplementary Fig. 3b), a phenomenon also observed in *Atg5^−/−^* cells^29^. We then interrogated mitochondrial function in *Redd1*^*−/−*^ cells. We observed substantially decreased basal O_2_ consumption, oxidative ATP generation and maximal respiratory capacity in these cells, consistent with profound mitochondrial dysfunction (Fig. 3f and Supplementary Fig. 3c). Notably, decreased oxidative ATP production in these cells is in keeping with the increase in glucose uptake, glycolysis and lactate production we previously observed in *Redd1*^*−/−*^ cells^20^. In order to test the capacity for oxidative ATP generation in these cells by an independent means we placed cells in galactose, which cannot be used to produce ATP through (non-oxidative) glycolysis. As predicted, *Redd1*^*−/−*^ cells demonstrated a substantial drop in ATP levels when placed in galactose, while wild-type cells maintained their ATP content (Fig. 3g). Taken together, these findings demonstrate a role for REDD1 in maintaining the autophagic flux and preserving mitochondrial homeostasis.

### REDD1-dependent ROS control autophagy through ATG4B activity

**Figure 4:**
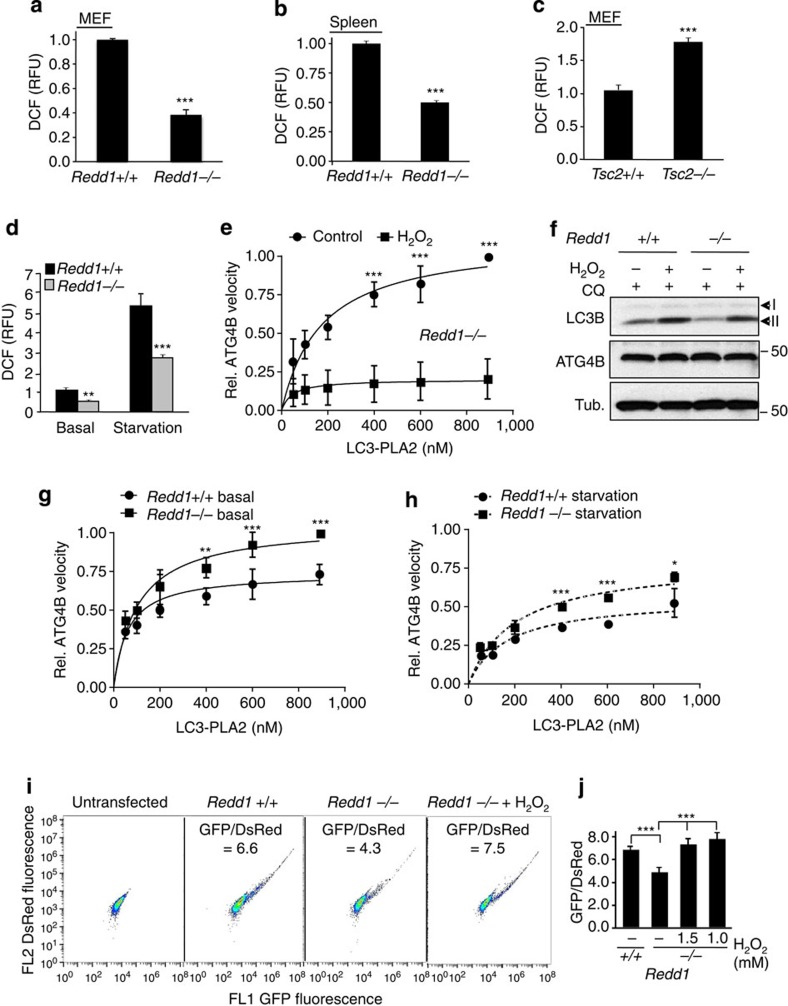
REDD1-dependent ROS controls autophagy and ATG4B activity. (**a**,**b**) Reduced H_2_O_2_ in *Redd1*^*−/−*^ MEFs and splenocytes from *Redd1*^*−/−*^ mice as compared with wild-type controls, assessed by staining with CM-H2DCFDA (3 μM) and then flow cytometry. Shown are the means from three independent experiments/mice measured in triplicate. (**c**) *Tsc2*^*−/−*^ MEFs exhibit elevated cytosolic H_2_O_2_. (**d**) Decreased H_2_O_2_ induction in *Redd1*^*−/−*^ versus wild-type cells on starvation (0.1% FBS). (**e**) Suppression of ATG4B activity by H_2_O_2_ (3 mM, 6 h) in *Redd1*^*−/−*^ cells, assessed by incubation of cellular extracts with the LC3-PLA_2_ substrate at the indicated concentrations. Each data point for velocity is derived from 60 sequential measurements, with the mean and 95% confidence intervals (shown as error bars) calculated by regression analysis (see Methods). (**f**) H_2_O_2_ (3 mM, 6 h) restores LC3B processing in *Redd1*^*−/−*^ cells to wild-type levels in the presence of CQ (30 μM, 4 h). Note similar levels of ATG4B in *Redd1*^*−/−*^ cells and wild-type cells in the absence or presence of H_2_0_2_. β-Tubulin (Tub.), loading control. (**g**) Elevated ATG4B activity in *Redd1*^*−/−*^ versus wild-type cells under basal conditions, assessed as described for **e**. (**h**) Persistent increase in ATG4B activity in *Redd1*^*−/−*^ versus wild-type cells under starvation (0.1% FBS, 24 h). (**i**) Enhanced cleavage of unprocessed LC3, as evidenced by a decreased GFP/DsRed ratio (the mean fluorescence of expressing cells) in *Redd1*^*−/−*^ compared with wild-type cells, transfected with double-tagged DsRed-LC3-GFP and analysed using flow cytometry. H_2_O_2_ (1 mM, 6 h) inhibits ATG4B to increase the GFP/DsRed ratio in *Redd1*^*−/−*^ cells. (**j**) The mean and s.d. of four independent experiments as in **i**, measured in triplicate. ***P<0.001, **P<0.01, *P<0.05, by paired *t*-test (**a**–**e**) or repeated measures ANOVA (**g**,**h**).

In order to unravel the mechanism of REDD1-mediated autophagy induction, we first interrogated the key physiological phenotypes associated with REDD1 loss. Previous work had linked REDD1 upregulation to the induction of cytosolic H_2_O_2_, a key ROS^17^. We therefore analysed cellular H_2_O_2_ levels in *Redd1*^*−/−*^ mice and found that indeed both *Redd1*^*−/−*^ cells and tissues exhibit dramatically lower total H_2_O_2_ levels than their wild-type counterparts (Fig. 4a,b). Notably, the opposite was true for *Tsc2*^*−/−*^ cells, which showed substantially elevated H_2_O_2_ levels, implying that decreased ROS in the absence of REDD1 was an mTORC1-independent phenotype (Fig. 4c). To determine whether ROS was a potential regulator of autophagy in this setting, we assessed H_2_O_2_ levels in response to starvation, given that starvation potently induces autophagy in the presence but not in the absence of REDD1 (Fig. 2a). Remarkably, starvation dramatically induced H_2_O_2_ levels in wild-type cells that temporally correlated with induction of autophagy in this setting (Fig. 4d). Most importantly, this oxidative burst was substantially attenuated in *Redd1*^*−/−*^ cells (Fig. 4d).

Given the reduced autophagosome size and number observed in the absence of REDD1, we next evaluated the potential effect of altered ROS on the enzymatic cascade involved in autophagosome assembly. In both yeast and mammals, the LC3B homologue is first cleaved by the ATG4 cysteine protease, and then conjugated to phosphatidylethanolamine by the ATG12–ATG5–ATG16L1 complex^21^. Phosphatidylethanolamine-conjugated LC3B is critical for elongation and closure of the developing autophagosome. ATG4 also plays a critical role in delipidation of LC3B, which results in LC3B redistribution out of the membrane and thus inhibits autophagosome maturation^30^. Intriguingly, the cysteine protease activity of ATG4 is thought to be regulated by ROS^31^. Low ROS levels are associated with a reduced state of the key cysteines and increased cleavage of the ATG4 substrate, while increased ROS levels are associated with decreased cleavage^31^. Thus, a model has been proposed whereby ROS is induced through an unknown mechanism, thereby attenuating ATG4 activity, blocking LC3B delipidation and promoting autophagosome maturation^30^,^31^,^32^.

We therefore hypothesized that REDD1 was a key mediator of this signalling pathway, and that decreased H_2_O_2_ resulting from REDD1 loss might promote hyperactivity of ATG4, thereby explaining defective autophagy in these cells. While four ATG4 orthologues are present in mammals, only genetic deletion of ATG4B results in an autophagy phenotype^33^. To assess ATG4B catalytic activity we turned to a novel and quantitative assaying involving a synthetic substrate, LC3B fused to the N terminus of phospholipase A_2_ (LC3B-PLA_2_), which on cleavage releases active PLA_2_ for fluorogenic assay^34^. In order to estimate *k*_cat_/*K*_m_ values for catalytic efficiency, we used regression analysis to fit a series of time-dependent fluorescence curves captured at different substrate concentrations^34^. As predicted, treatment with exogenous H_2_O_2_ itself substantially inhibited ATG4B activity (Fig. 4e), and ATG4B inhibition by H_2_O_2_ was sufficient to induce the appearance of lipidated LC3 in wild-type cells (Fig. 4f and Supplementary Fig. 4a).

In keeping with our hypothesis, we observed a striking and consistent elevation in the enzymatic activity of ATG4B in *Redd1*^*−/−*^ versus wild-type cells, with calculated *k*_cat_/*K*_m_ values of 8.7 × 10^5^ versus 4.8 × 10^5^ M^−1^ s^−1^, respectively (Fig. 4g). Furthermore, suppression of ATG4B activity by H_2_O_2_ in *Redd1*^*−/−*^ cells was sufficient to restore basal LC3 lipidation to wild-type levels (Fig. 4f). Of note, no differences were observed in levels of ATG4B, or in other ATG4 family members, between wild-type and *Redd1*^*−/−*^ cells, either in the presence or absence of H_2_O_2_ (Fig. 4f and Supplementary Fig. 4b). Concordant with the induction of ROS following starvation and its attenuation in *Redd1*^*−/−*^ cells (Fig. 4d), ATG4B activity was reduced by starvation in wild-type cells (Supplementary Fig. 4c) but remained elevated in *Redd1*^*−/−*^ cells under these conditions (Fig. 4h).

In order to corroborate these findings we tested ATG4B enzymatic activity in these cells by an independent method. We introduced a well-characterized, double-fluorescence-tagged, unprocessed LC3 protein (DsRed-LC3-GFP) that includes the ATG4B recognition site for cleavage of pro-LC3 (ref. 35). Protein expression can thus be monitored quantitatively by fluorescence intensity, and ATG4B-dependent LC3 cleavage leads to degradation of the C-terminal GFP resulting in a decreased GFP/DsRed ratio (Supplementary Fig. 4d). Importantly, the specificity of this construct and the GFP/DsRed shift for ATG4B has been demonstrated, as a stress-induced shift in GFP/DsRed was abrogated following deletion of a five-amino-acid recognition site for ATG4B in LC3 (ref. 35). Our data above predict that the GFP/DsRed ratio should be decreased in the absence of REDD1 even under basal (non-stressed) conditions owing to elevated ATG4B activity. Indeed, a decreased GFP/DsRed ratio was consistently observed in *Redd1*^*−/−*^ compared with wild-type cells expressing this construct (Fig. 4i,j). Furthermore, the addition of ROS was sufficient to restore the GFP/DsRed ratio to wild-type levels in *Redd1*^*−/−*^ cells (Fig. 4i,j) in keeping with the ability of ROS to suppress endogenous ATG4B activity (Fig. 4e). Taken together, these findings define REDD1 as a key endogenous regulator of ATG4B activity, LC3B processing and autophagy through control of ROS.

### A REDD1/TXNIP complex controls ROS, ATG4B and autophagy

**Figure 5:**
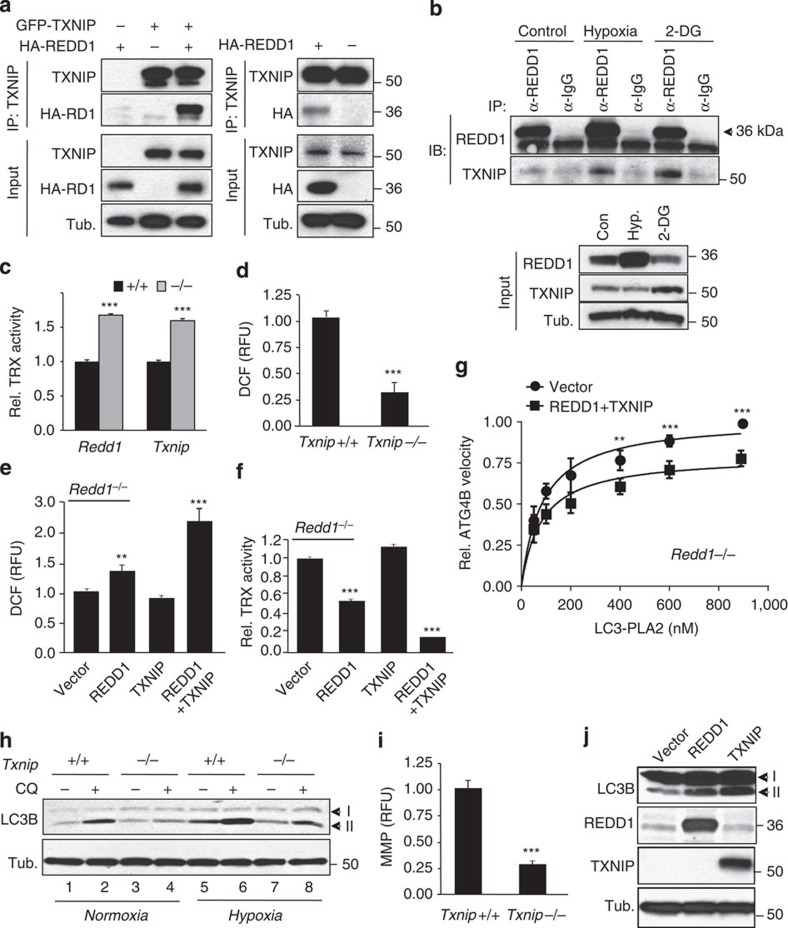
REDD1 and TXNIP interact to control ROS, ATG4B and autophagy. (**a**) Physical association of REDD1 with both transfected GFP-TXNIP (left) and endogenous TXNIP (right) following transfection of HA-REDD1 and IP with α-TXNIP in 293T cells. (**b**) The endogenous REDD1/TXNIP complex is induced by hypoxia (1% O_2_, 16 h) and energy stress (2-deoxyglucose (2-DG), 20 mM, 16 h) in 293T cells. Lysates were subjected to IP with α-REDD1 antibody. (**c**) Increased thioredoxin (TRX) antioxidant activity in *Redd1*^*−/−*^ and *Txnip*^*−/−*^ cells, measured by reduction of insulin disulfides. Shown is the mean of three independent experiments performed in triplicate. (**d**) Reduced H_2_O_2_ in *Txnip*^*−/−*^ MEFs stained with CM-H2DCFDA (3 μM) followed by flow cytometry. (**e**) TXNIP fails to induce H_2_O_2_ in *Redd1*^*−/−*^ cells unless co-transfected with REDD1. (**f**) Co-transfection of REDD1 and TXNIP potently inhibits TRX activity in *Redd1*^*−/−*^ cells. (**g**) Co-transfection of REDD1 and TXNIP is sufficient to suppress ATG4B activity in *Redd1*^*−/−*^ cells, assessed as in Fig. 4. (**h**) Impaired autophagy under basal and hypoxic (1% O_2_, 4 h) conditions in *Txnip*^*−/−*^ MEFs, shown in the absence or presence of CQ (30 μM, 4 h). (**i**) Reduced MMP in *Txnip*^*−/−*^ cells, assessed by staining with TMRE (100 nM) followed by flow cytometry. (**j**) Expression of REDD1 or TXNIP is sufficient to induce autophagy in transfected 293T cells. ***P<0.001, **P<0.01, by paired *t*-test (**c**–**f**, **i**) or repeated measures ANOVA (**g**).

While these findings point to REDD1-regulated ROS as the crucial signalling intermediates for autophagy regulation, we had yet to determine how REDD1 regulates cellular ROS levels. An initial clue was provided by a recent report of the phenotype of mice lacking the pro-oxidant protein TXNIP^36^. Cells from *Txnip*^*−/−*^ animals exhibit reduced oxidative phosphorylation, mitochondrial ultrastructural derangements and enhanced glycolysis reminiscent of *Redd1*^*−/−*^ cells^36^. We therefore tested the possibility of a physical interaction between REDD1 and TXNIP^37^. Indeed, in co-immunoprecipitation (co-IP) studies involving either co-transfection or single transfection, REDD1 and TXNIP formed a robust complex that was resistant even to harsh detergent conditions (Fig. 5a). Furthermore, endogenous REDD1 and TXNIP were readily observed to be associated by co-IP (Fig. 5b). Most importantly, the endogenous REDD1/TXNIP complex was induced under conditions of hypoxia and energy stress (Fig. 5b)^11^,^18^,^38^,^39^,^40^. Thus, endogenous REDD1 and TXNIP are induced and form a robust physical complex in response to cellular stress.

These findings collectively led us to ask whether REDD1 is required for TXNIP pro-oxidant function. TXNIP, a member of the α-arrestin protein family, is the major endogenous inhibitor of the antioxidant thioredoxins (TRX1/2), forming a mixed disulphide with TRX proteins and thereby blocking their reducing ability^41^. As shown in prior reports we found that TRX activity, measured as thioredoxin reductase-dependent reducing capacity, was substantially increased in *Txnip*^*−/−*^ cells (Fig. 5c)^36^. We found that in *Redd1*^*−/−*^ cells TRX activity was similarly elevated (Fig. 5c). In keeping with this increased TRX activity, the dramatically reduced cytosolic H_2_O_2_ levels seen in *Redd1*^*−/−*^ cells were recapitulated in *Txnip*^*−/−*^ cells (Fig. 5d). We then directly assessed the requirement for REDD1 in TXNIP function. Remarkably, transfection of TXNIP into *Redd1*^*−/−*^ cells had no effect on cellular ROS levels; REDD1 reconstitution alone modestly induced ROS, while co-transfection of both proteins robustly induced ROS (Fig. 5e). Importantly, these effects on ROS were mirrored by inverse effects on TRX activity (Fig. 5f). Thus, REDD1 is required for TXNIP pro-oxidant function.

On the basis of these findings we next asked whether reconstitution of the REDD1/TXNIP complex into *Redd1*^*−/−*^ cells is sufficient to inhibit ATG4B catalytic activity. In keeping with the potent induction of ROS we observed in this setting, we measured a highly significant suppression of ATG4B activity following REDD1/TXNIP expression (Fig. 5g). In order to corroborate the contribution of TXNIP to autophagy we then assessed whether *Redd1*^*−/−*^ and *Txnip*^*−/−*^ cells share common phenotypes in autophagy regulation. Strikingly, we found that endogenous TXNIP was required for autophagy, demonstrating for the first time that *Txnip*^*−/−*^ cells, similar to *Redd1*^*−/−*^ cells, are defective in both basal and hypoxia-induced autophagy (Fig. 5h and Supplementary Fig. 5). As in *Redd1*^*−/−*^ cells, defective autophagy was associated with abnormally depolarized mitochondria (Fig. 5i)^36^. In addition, overexpression of REDD1 or TXNIP was sufficient to induce autophagy (Fig. 5j). Collectively, these data reveal a new mechanism of autophagy regulation: the REDD1/TXNIP complex is both necessary and sufficient for ROS induction in response to stress, which suppresses the activity of ATG4B, blocks LC3B delipidation and promotes autophagosome maturation and autophagy.

### Failed autophagy impedes exercise capacity in *Redd1* ^−/−^ mice

**Figure 6:**
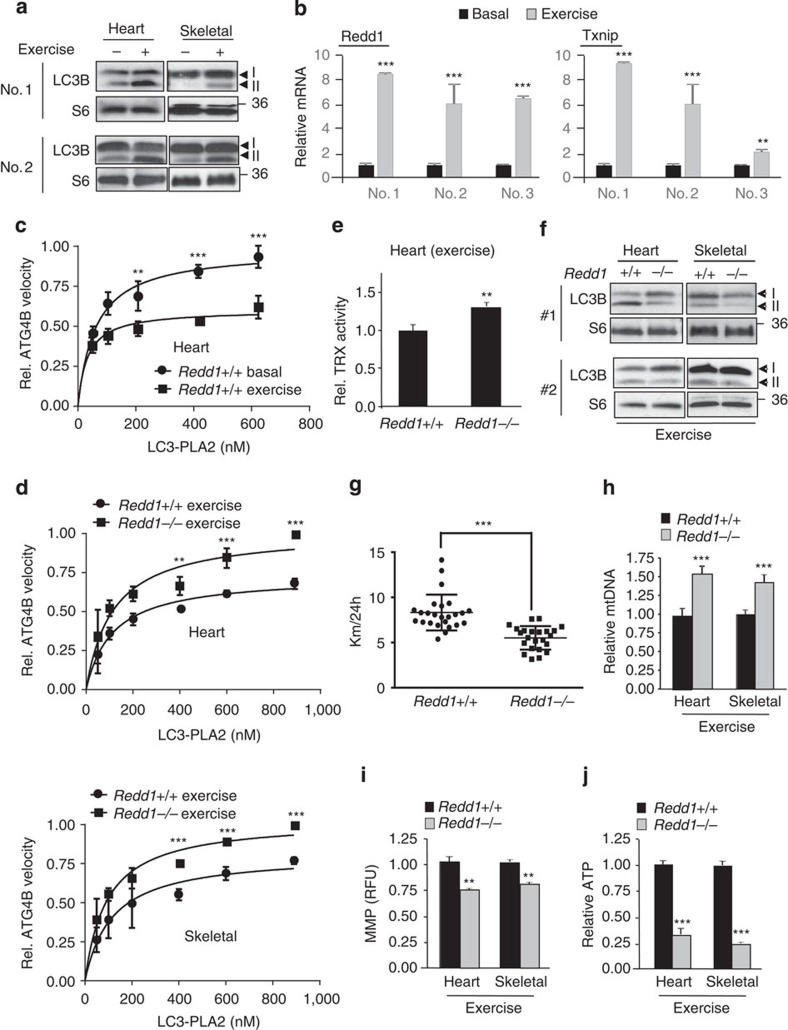
Impaired ROS/ATG4B/autophagy compromises exercise capacity in *Redd1*^*−/−*^ mice. (**a**) Exercise (treadmill run, 2 h) induces autophagy in the muscle of wild-type mice, as assessed by LC3B processing. RPS6 (S6) protein, loading control. Duplicate panels correspond to individual mice. (**b**) REDD1 and TXNIP mRNA induction by exercise (treadmill run, 2 h) in the skeletal muscle of wild-type mice, measured using QRT–PCR relative to β-actin. Numbers refer to individual mice. (**c**) Exercise suppresses ATG4B activity in the heart of wild-type mice, assessed as in Fig. 4. Shown are the mean values from three mice per condition. (**d**) Hyperactivity of ATG4B in the heart and skeletal muscle of treadmill-exercised *Redd1*^*−/−*^ mice, assessed and quantitated as in **c**. (*N*=3 mice per genotype.) (**e**) Increased TRX activity in the heart of treadmill-exercised *Redd1*^*−/−*^ mice, measured as in Fig. 5. (*N*=3 mice per genotype.) (**f**) Impaired autophagy induction by treadmill exercise in the muscle of *Redd1*^*−/−*^. Duplicate panels correspond to individual mice. (**g**) Decreased exercise capacity in *Redd1*^*−/−*^ mice (voluntary running wheel, 24 h). Each point represents an individual run; eight mice per genotype were subjected to at least three runs each; error bars, s.e.m. (**h**) Increased mitochondrial (mt) number in the muscle of treadmill-exercised *Redd1*^*−/−*^ compared with wild-type mice. (*N*=3 mice per genotype.) (**i**) Reduced MMP in tissues as in **h**, assessed in cell-free mitochondrial fractions. (**j**) ATP concentration measured in lysates of tissues described in **h**. Except as noted, all error bars indicate s.d. ***P<0.001, **P<0.01, by paired *t*-test (**b**,**e**,**h**,**j**), or repeated measures ANOVA (**c**,**d**), or Mann–Whitney *U*-test (**g**).

Autophagy has recently been recognized as essential for maintaining exercise capacity, as reduced capacity is a common phenotype shared by several mouse genetic models of defective autophagy^42^,^43^,^44^. Consistent with these reports, we observed that forced exercise using a standard treadmill protocol robustly induced autophagy in several tissues of wild-type mice including heart and skeletal muscle (Fig. 6a)^44^. Concurrently, we found that exercise induced expression of both REDD1 and TXNIP in these same muscle tissues (Fig. 6b). As predicted, REDD1 and TXNIP induction by exercise was accompanied by substantial suppression of endogenous ATG4B enzymatic activity in these tissues (Fig. 6c). In both heart and skeletal muscle tissues of *Redd1*^*−/−*^mice, however, ATG4B activity remained abnormally elevated under exercise (Fig. 6d), and this activity was associated with defective TXNIP-dependent ROS as measured by TRX activity (Fig. 6e). Consequently, induction of autophagy by exercise was substantially attenuated in both heart and skeletal muscle of *Redd1*^*−/−*^ mice (Fig. 6f and Supplementary Fig. 6). Reflecting the physiologic importance of this defect, we found that failed autophagy induction was associated with a consistent and substantial reduction in capacity for sustained exercise in *Redd1*^*−/−*^ compared with wild-type mice (Fig. 6g).

**Figure 7:**
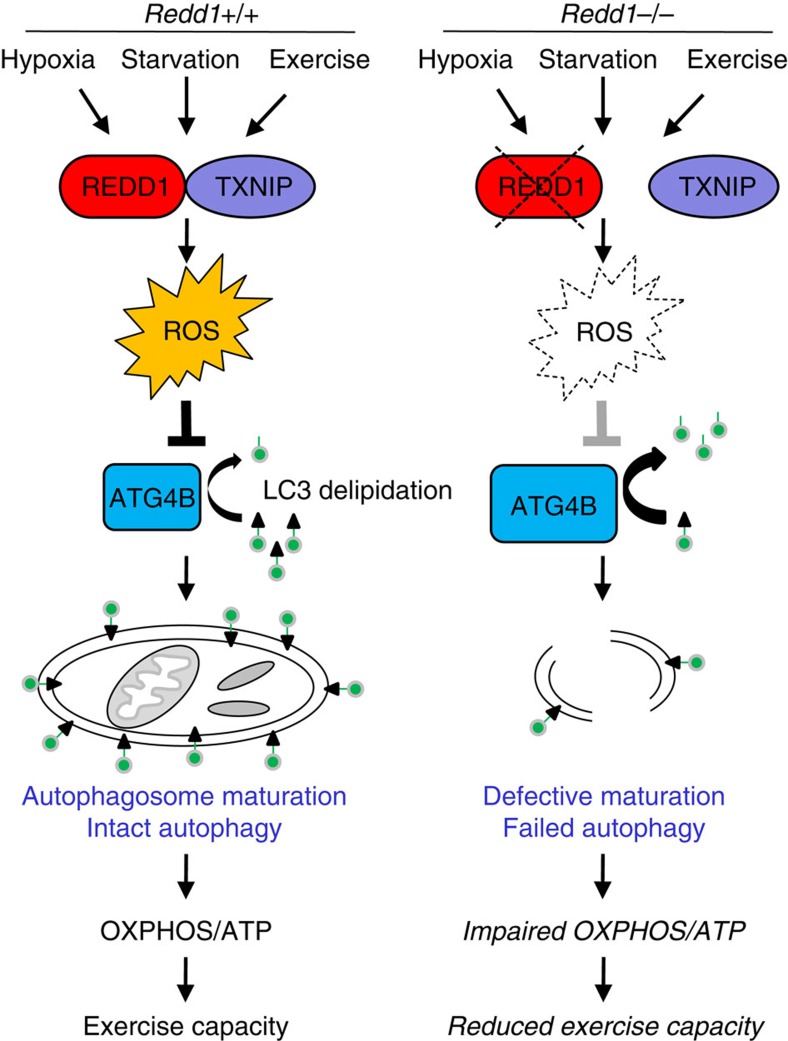
REDD1 controls stress-induced autophagy and energy homeostasis. REDD1 and TXNIP are upregulated by physiologic stress, forming a protein complex required for induction of ROS that inhibit ATG4B-mediated LC3 delipidation and thereby promote autophagosome maturation. In the absence of REDD1, decreased ROS results in hyperactivity of ATG4B and disabled autophagy, leading to accumulation of aged, dysfunctional mitochondria and defective oxidative metabolism that compromises ATP generation and exercise capacity.

Finally, we explored the specific link between defective autophagy and exercise capacity in these mice. We hypothesized a failure of bioenergetics, given the accumulation of abnormal mitochondria and disabled oxidative phosphorylation we had documented in the absence of REDD1-dependent autophagy (Fig. 3). Indeed, we observed both an increased number and depolarization of mitochondria in heart and skeletal muscle of *Redd1*^*−/−*^ compared with wild-type mice, which persisted following exercise (Fig. 6h,i). These mitochondrial abnormalities were associated with a striking deficit in ATP concentration of ∼30% of wild-type levels in muscle tissues of *Redd1*^*−/−*^ mice following forced exercise (Fig. 6j). All together, these results define a physiologic pathway for stress-induced ROS through REDD1/TXNIP that controls ATG4B activity to promote autophagy required for metabolic homeostasis (Fig. 7).

## Discussion

The process of macroautophagy is now established to play a fundamental role in organismal homeostasis under multiple stress states including starvation and exercise^43^,^44^,^45^,^46^. Our findings reveal a new and essential mechanism by which stress signals are transduced to control autophagy through induction of the metabolic regulator REDD1. We find that *Redd1*^*−/−*^ mice exhibit dramatic defects in autophagy, evidenced most clearly by reduced LC3B lipidation and degradation, and a decreased size and number of autophagic vesicles observed in their cells and tissues (Fig. 1). We demonstrate that this mTORC1-independent autophagy pathway controlled by REDD1 involves redox-dependent regulation of a key enzyme in autophagosome assembly and maturation, the cysteine protease ATG4B. *Redd1*^*−/−*^ cells exhibit dramatically decreased basal ROS and increased ATG4B enzymatic activity, and they fail to induce ROS and suppress ATG4B activity appropriately in response to stress. As predicted by this model, H_2_O_2_ is sufficient to suppress ATG4B activity and to rescue the LC3B lipidation defect in REDD1^*−/−*^ cells (Fig. 4). We further reveal that REDD1-dependent ROS induction is mediated via its interaction with TXNIP, a central pro-oxidant factor that we find is functionally disabled in the absence of REDD1. Overexpression of the REDD1/TXNIP complex recapitulates the physiologic induction of ROS, suppression of ATG4B and induction of autophagy induced by cellular stress (Fig. 5).

Our model proposes that defective LC3B processing is the basis for the *Redd1*^*−/−*^ phenotype. Support for the physiologic validity of this model is provided by common phenotypic features observed between *Redd1*^*−/−*^ mice and mice lacking LC3B itself. Tissues of both *Redd1*^*−/−*^ and *Lc3b*^*−/−*^ mice do not lack autophagosomes entirely, but instead exhibit a reduced number of undersized autophagic vesicles on stress (Fig. 1)^47^. Intriguingly, CS-induced lung damage has been linked to activation of autophagy^48^, and both *Redd1*^*−/−*^ and *Lc3b*^*−/−*^ mice exhibit a dramatic protection against such damage, including significantly decreased levels of apoptosis in the lungs after CS exposure, and resistance to CS-induced emphysema^16^,^47^. Consistent with a common mechanism, in both cases these phenotypes are linked to defects in NF-kB signalling, potentially implying a specific role for REDD1-dependent autophagy in this pathway^49^,^50^.

REDD1 is established to function as an upstream inhibitor of TORC1 activity through the TSC1/2 complex in flies and mammals^10^,^11^,^14^. Here we have discovered that, in addition, REDD1 is essential for induction of autophagy through a mechanism downstream and independent of mTORC1 (Fig. 3). These findings therefore provide a compelling example of how a single effector coordinates mTORC1/autophagy signalling at multiple levels. Presumably, this synchronization is critical for balancing the energy-intensive, anabolic effects of mTORC1 activation such as enhanced protein translation, with autophagy-induced catabolic recycling of cellular components to generate energy and building blocks. Similar to REDD1, the energy stress kinase AMPK also inhibits mTORC1 upstream (in part through TSC1/2) while at the same time promoting mTORC1-independent autophagic membrane assembly, in this case through phosphorylation of substrates downstream of mTORC1 (ref. 8). Similarly, the prominent autophagy substrate p62 (sequestosome 1), which is induced under conditions of high nutrients and accumulates in response to decreased autophagy, functions itself to activate mTORC1 and thereby control autophagy^51^. Taken together, these findings support the conclusion that tight coordination between mTORC1 activity and the autophagic flux is an evolutionary imperative.

ROS is now known to function as the key intracellular signalling molecules, yet in most cases the specific mechanisms and targets of ROS regulation remain undefined. Here we reveal that a complex between REDD1 and the pro-oxidant protein TXNIP mediates a stress-induced oxidative burst that controls ATG4B activity and thereby LC3B processing. Our finding that low H2O2 levels in the absence of REDD1 are associated with increased ATG4B activity is fully in keeping with prior work showing that reducing agents promote ATG4 substrate cleavage^31^. However, our work is the first to reveal the endogenous pro-oxidant signalling mechanism that regulates ROS to control this critical enzyme. While the precise mechanism by which REDD1 controls TXNIP-dependent pro-oxidant function remains to be elucidated, the remarkable shared phenotypes of *Redd1*^*−/−*^ and *Txnip*^*−/−*^ cells that we have defined strongly support a common functional pathway for autophagy regulation. These include increased TRX reducing capacity and cytosolic ROS, defective basal and stress-induced autophagy, and accumulation of depolarized, dysfunctional mitochondria (Fig. 5)^36^. In addition, a recent report describes a comparable defect in exercise capacity of *Txnip*^*−/−*^ mice as we observe for *Redd1*^*−/−*^ mice^52^. Nevertheless, it is not expected that *Redd1^−/−^* and *Txnip^−/−^* will be exact phenocopies, given that TXNIP contributes to processes such as cellular glucose uptake through multiple, apparently redox-independent mechanisms^53^.

Finally, our work forges a new and specific mechanistic link between redox-dependent signalling and exercise physiology. ROS has been associated in some contexts with enhanced exercise capacity through enhanced mitochondrial biogenesis^54^, while increased ROS has also been associated with muscle wasting, mitochondrial damage and apoptotic signalling^55^. We have discovered a REDD1-mediated ROS signal transduction mechanism required for efficient muscle ATP generation and exercise capacity via autophagy and mitochondrial homeostasis. A central role for muscle-specific autophagy failure in the exercise phenotype of *Redd1*^*−/−*^ mice is supported by the defective oxidative metabolism and compromised exercise capacity observed in mice with muscle-specific deletion of *Atg7* (ref. 28). As with loss of Atg7, depolarized mitochondria and defective oxidative ATP generation are in fact hallmarks of *Redd1*^*−/−*^ cells and tissues. A contribution of dysfunctional mitochondria to disabled autophagy in the absence of *Redd1* cannot entirely be ruled out. However, our finding that *Redd1*^*−/−*^ mitochondria emit abnormally increased ROS^20^, together with our detailed demonstration of how a decrease in cytosolic ROS deregulates autophagy in this setting, supports our model (Fig. 7). Notably, decreased mitochondrial function is a common trait observed in chronic muscle disuse associated with aging and with some muscle-wasting syndromes^55^. It is therefore conceivable that the molecular pathway defined herein may provide new targets for therapeutic intervention aimed at improving oxidative metabolism and muscle function under such conditions.

## Methods

### Tissue culture

Primary and immortalized wild-type, *Redd1*^*−/−*^ and *Txnip*^*−/−*^ MEFs as well as U2OS human osteosarcoma cells expressing tetracycline-regulated wild-type REDD1 and REDD1-RPAA were maintained in DMEM supplemented with 10% fetal bovine serum (FBS), penicillin and streptomycin. Cells were transfected with GFP-LC3, REDD1 and TXNIP expression constructs using Fugene 6.

### QRT–PCR and mtDNA number analysis

Total RNA extraction, cDNA synthesis and QRT–PCR were performed using five-point standard curves to assign relative gene expression values^20^. The expression of each gene was normalized to *β-actin*. Primers used are as follows: *Redd1*: 5′-GACAGCAGCAACAGTGGCTTC-3′; 5′-CCACGCTATGGCAGCTCTTGC-3′; *Txnip:* 5′-CTGCAGAAGATCAGACCATCC-3′; 5′-AGCCAGGGACACTGACGTA-3′; *Actb*: 5′-CCAACCGTGAAAAGATGACC-3′; 5′-CCAGAGGCATACAGGGACAG-3′. Mitochondrial number was measured using primers for the mitochondrial-encoded *COXII* gene normalized to the nuclear-encoded *β-globin* gene of isolated genomic DNA from cells and tissues. Primers used are as follows: *Mt-Co2*: 5′-GCCGACTAAATCAAGCAACA-3′; 5′-CAATGGGCATAAAGCTATGG-3′*; β-globin:* 5′-GAAGCGATTCTAGGGAGCAG-3′; 5′-GGAGCAGCGATTCTGAGTAGA-3′.

### Co-IP and western blot analysis

For co-IP studies, cell extracts were prepared in lysis buffer (50 mM Tris-HCl pH 8.0, 2 mM EDTA, 250 mM NaCl and 0.1% Triton X-100) containing protease inhibitors (Roche) for 50 min on ice. Cellular debris was clarified; next supernatant was pre-cleared with 30 μl protein G-agarose beads for 1 h at 4 °C, followed by incubation with antibodies for 2 h and then pull downed with 30 μl protein G-agarose beads for 2 h or overnight. Beads were washed three times with lysis buffer and proteins were eluted with 40 μl of 2 × SDS–PAGE sample buffer, boiled for 5 min and analysed by western blot analysis. For evaluation of LC3B, total protein extracts were prepared using RIPA buffer (150 mM NaCl, 50 mM Tris-HCl pH 8.0, 1.0% NP40, 0.5% sodium deoxycholate and 0.1% SDS) containing protease inhibitor (Roche) and phosphatase inhibitor cocktails (Sigma). Equal amounts of lysates were subjected to SDS–PAGE. Blots were incubated with antibodies recognizing the following proteins: LC3B (Cell Signaling, no. 2775), β-tubulin (Millipore MAB 3408), p62 (Cell Signaling no. 5114), TXNIP (MBL Laboratories K0204-3 and Santa Cruz Biotechnologies sc-33099), REDD1 (Bethyl Laboratories A302-169A and Santa Cruz Biotechnologies sc-46034), phospho-S6 S235/S236 (Cell Signaling no. 2211), total S6 (Cell Signaling no. 2217), phospho-S6 Kinase T389 (Cell Signaling no. 9205), Hypoxia-inducible factor 1-α (HIF-1α) (Novus Biologicals NB100-449), ATG4B (Santa Cruz Biotechnologies sc-130968), haemagglutinin (HA) epitope (Covance MMS-101R) and TOM20 (Santa Cruz Biotechnologies sc-11415). All antibodies were used at a 1:1,000 dilution from the commercial stock. All western analyses were repeated in at least three independent experiments.

### Flow cytometric analysis and mitochondrial fractionation

MEFs and dissociated splenocytes from wild-type and *Redd1*^*−/−*^ mice were stained for 45 min in DMEM with 3 μM CM-H2DCFDA (Invitrogen) to assess cytosolic H_2_O_2_, 5 μM MitoSox Red (Invitrogen) to assess mitochondrial superoxide, 100 nM Tetramethylrhodamine, Ethyl Ester (Invitrogen) to assess mitochondrial membrane potential and 100 nM MitoTracker Green FM (Invitrogen) to assess mitochondrial mass. To evaluate autophagic activity in live cells, GFP-LC3 was retrovirally transduced into cells. Forty-eight hours later cells were cultured under normoxia or hypoxia (1% O_2_) for 4 h and then analysed. To examine glucose uptake, 100 μM of the fluorescent glucose analogue 2-NBDG (Invitrogen) was added to cell culture media 16 h before analysis. For cell-free mitochondrial preparations, cells/tissues were suspended in IBc buffer (200 mM sucrose, 10 mM Tris-3-(*N*-morpholino)propanesulfonic acid (MOPS), 1 mM EGTA/Tris, pH 7.4) followed by dounce homogenizing and then centrifuging at 1,900 r.p.m. to pellet and remove nuclear and cellular debris. Supernatant was then collected, centrifuged at 10,000 r.p.m. to pellet mitochondrial and resuspended in IBc buffer^56^. Mitochondrial size was determined by measuring the forward side scatter using a FACSCalibur (BD Biosciences).

### Transmission electron and confocal microscopy

For TEM, cells and tissues were immediately fixed with 2.0% glutaraldehyde in 0.1 M sodium cacodylate buffer, pH 7.4 (Electron Microscopy Sciences) and then post-fixed in 1% osmium tetroxide, dehydrated in ethanol and embedded in epon. Ultrathin sections were collected on formvar-coated grids and stained with uranil acetate and lead citrate and then samples were examined with a JEOL JEM 1011 transmission electron microscope. Confocal images of LC3-stained cells were captured using a scanning laser confocal microscope (Nikon Eclipse Ti; Nikon) using × 40 or × 60 oil lenses and NIS-Elements software (Nikon). For quantification of endogenous LC3 puncta number, the red channel of the RGB images from CQ-treated cells were extracted and converted to greyscale images by auto-thresholding of the custom-written ImageJ macro conning plug-ins. At least 60 cells per sample in three to five images were analysed for number and for size using the ‘Analyze particles' function of the software^57^. Microscopy studies were repeated in at least three independent experiments, and images shown are representative.

### Oxygen consumption and mitochondrial oxidative phosphorylation and cellular ATP analysis

The cellular oxygen consumption rate of live cells was assessed using the Seahorse XF24 (Seahorse Biosciences). Respiration was measured under basal condition as well as in the presence of mitochondrial inhibitor 0.25 μM oligomycin, 5 μM uncoupler carbonyl cyanide-4-(trifluoromethoxy)phenylhydrazone (FCCP) and 1 μM respiratory chain inhibitors antimycin A and rotenone. To assess mitochondrial oxidative phosphorylation capacity, cells were cultured in glucose-free DMEM supplemented with 1 mM sodium pyruvate, 10% dialysed FBS and 25 mM glucose or galactose for 48 h. Cells were trypsinized and counted. ATP content of cells was determined using the Cell Titer-Glo kit (Promega).

### Endogenous ATG4B and thioredoxin activity

Cells were plated and cultured under basal condition with 10% FBS or under starvation with 0.1% FBS overnight, resuspended in lysis buffer (25 mM Tris-HCl, pH 8.0, 100 mM NaCl, 1 mM CaCl_2_, 5 mM MgCl, 5% Glycerol, 0.1% NP-40 and protease inhibitors) and then sonicated. Lysates were clarified using centrifugation and cellular ATG4B activity was assessed by incubation of equivalent lysate amounts with various concentrations of LC3B-PLA2 fusion protein substrate (25–890 nM) in reaction buffer (20 mM Tris-HCl, pH 8.0, 2 mM CaCl and 20 μM NBD-C_6_-HPC; Invitrogen). Fluorescence intensity was measured for 2 h at 2-min intervals (60 measurements per data point) using a SpectraMax 5 M plate reader (Molecular Devices) at room temperature with excitation and emission wavelength of 485 nm and 530 nm, respectively. To assess catalytic efficiency, the *k*_cat_/*K*_m_ of Atg4B on LC3B-PLA2 was determined using nonlinear regression fitting of a series of progress curves captured at different substrate concentrations using the established sequential activation model^34^. *P* values for differences between velocity at each substrate concentration were determined by multiple-measures analysis of variance (ANOVA). Thioredoxin activity was assessed using the insulin disulfide reduction assay^36^,^58^.

### Unprocessed LC3 cleavage assay

MEFs were transfected with pQCXI Puro expressing the dual-tagged DsRed-LC3-GFP protein (A gift from David Sabatini; Addgene plasmid no. 31182) using FuGENE 6 (Promega)^35^. Twenty-four hours post transfection, cells were harvested and analysed using flow cytometry. The mean fluorescence intensity of the transfected populations was determined using mock-transfected cells as controls. Cleavage of unprocessed LC3 by ATG4B is measured by the mean fluorescence intensity ratio of GFP/DsRed (Supplementary Fig. 4d)^35^.

### Treadmill and voluntary running wheel

All animal studies were conducted according to the protocols approved by the accredited MGH Subcommittee on Research Animal Care. In both exercise modalities, age- and sex-matched C57BL/6 mice (8- to 14-week-old) were used. For treadmill, mice were acclimatized on an Exer 6 treadmill (Columbus Instruments) for 2 days (day 1 for 5 min at 8 m min^−1^ and day 2 for 5 min at 8 m min^−1^ followed by another 5 min at 10 m min^−1^). On day 3, mice were subjected to treadmill starting with a speed of 10 m min^−1^, and after 40 min, the speed was increased by a rate of 1 m min^−1^ every 10 min for 30 min, and then increased by a rate of 1 m min^−1^ every 5 min until mice reached exhaustion (≥5 s on the electric shocker without attempts to run)^44^. For voluntary running wheel, mice were individually placed in cages with a running wheel (Lafayette Instrument Co.) and the distance covered within a 24-h period was measured.

### Statistical analysis

Comparisons of individual data sets were performed in most cases using Student's *t*-test, and data are presented as the mean±ss.d. *P* values for differences between ATG4B velocity at each substrate concentration were determined by repeated-measures ANOVA. *P* values for exercise wheel analysis were generated by Mann–Whitney–Wilcoxon text. A *P* value of <0.05 was considered statistically significant.

## Author contributions

S.Q., M.D., X.S., D.D.V., D.S. and Z.N. performed experiments and data analysis. Z.Y., M.L., J.Y., S.-I.M., O.S.S., R.T.L. and J.C.R. provided the key reagents and experimental advice. L.W.E. conceived the project, designed experiments, analysed the data and wrote the manuscript. All authors reviewed and approved the manuscript.

## Additional information

**How to cite this article:** Qiao, S. *et al.* A REDD1/TXNIP pro-oxidant complex regulates ATG4B activity to control stress-induced autophagy and sustain exercise capacity. *Nat. Commun.* 6:7014 doi: 10.1038/ncomms8014 (2015).

## Supplementary Material

Supplementary Information Supplementary Figures 1-7
