# Supplementary material for: A REDD1/TXNIP pro-oxidant complex regulates ATG4B activity to control stress-induced autophagy and sustain exercise capacity
Source: Nat Commun. 2015 Apr 28;6:7014. doi: 10.1038/ncomms8014 (PMC4421852; doi:10.1038/ncomms8014)

Supplementary Figure 1 - Ellisen et al.

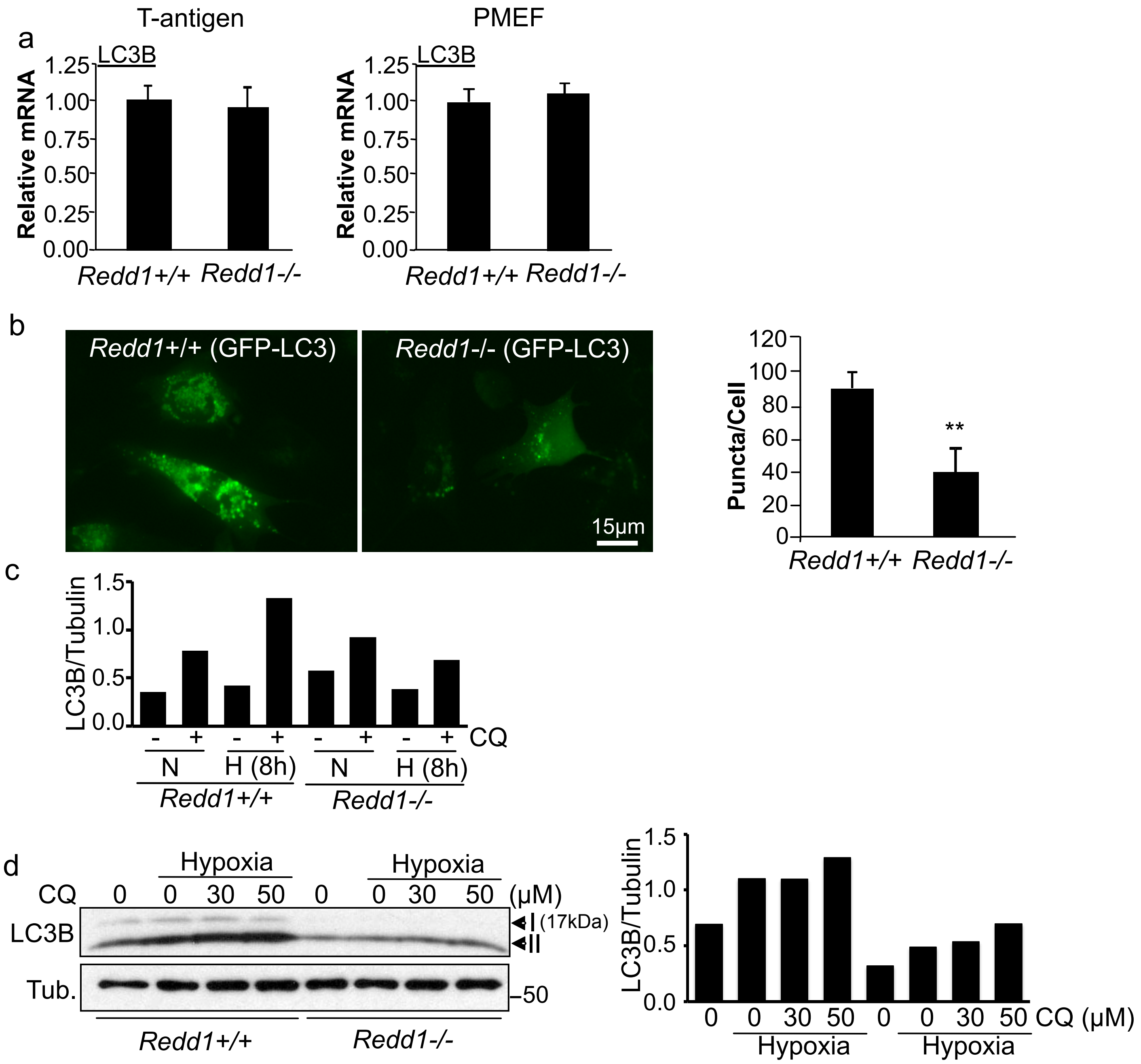


**Supplementary Figure 1. Defective autophagy in *Redd1*$^{-/-}$ cells.** (A). Reduced LC3B processing is not attributable to differences in LC3B mRNA expression between wild-type and *Redd1*$^{-/-}$ immortalized (left) and primary MEFs (right) as assessed by QRT-PCR. Error bars show SD of three measurements from experiments performed in duplicate. (B). Representative immunofluorescence images showing decreased GFP-tagged LC3 puncta in *Redd1*$^{-/-}$ cells as compared to wild-type cells cultured under basal conditions in the presence of CQ (30μM, 4h). N ≥ 20 cells counted per genotype. Scale bar, 15μm. (C). Densitometry analysis for Figure 1c. Bars show intensity of LC3B bands relative to β-Tubulin loading control. (D). Defective hypoxia-induced processing of LC3B in *Redd1*$^{-/-}$ cells. Cells were cultured hypoxia (1% $O_2$, 4h) and treated with the indicated concentrations of chloroquine (CQ, 4h). This experiment provides additional supportive data to Fig. 1c. Densitometry analysis is shown on the right panel. All error bars indicate SD. **P < 0.01 by paired t test (A, B)

Supplementary Figure 2 - Ellisen et al.

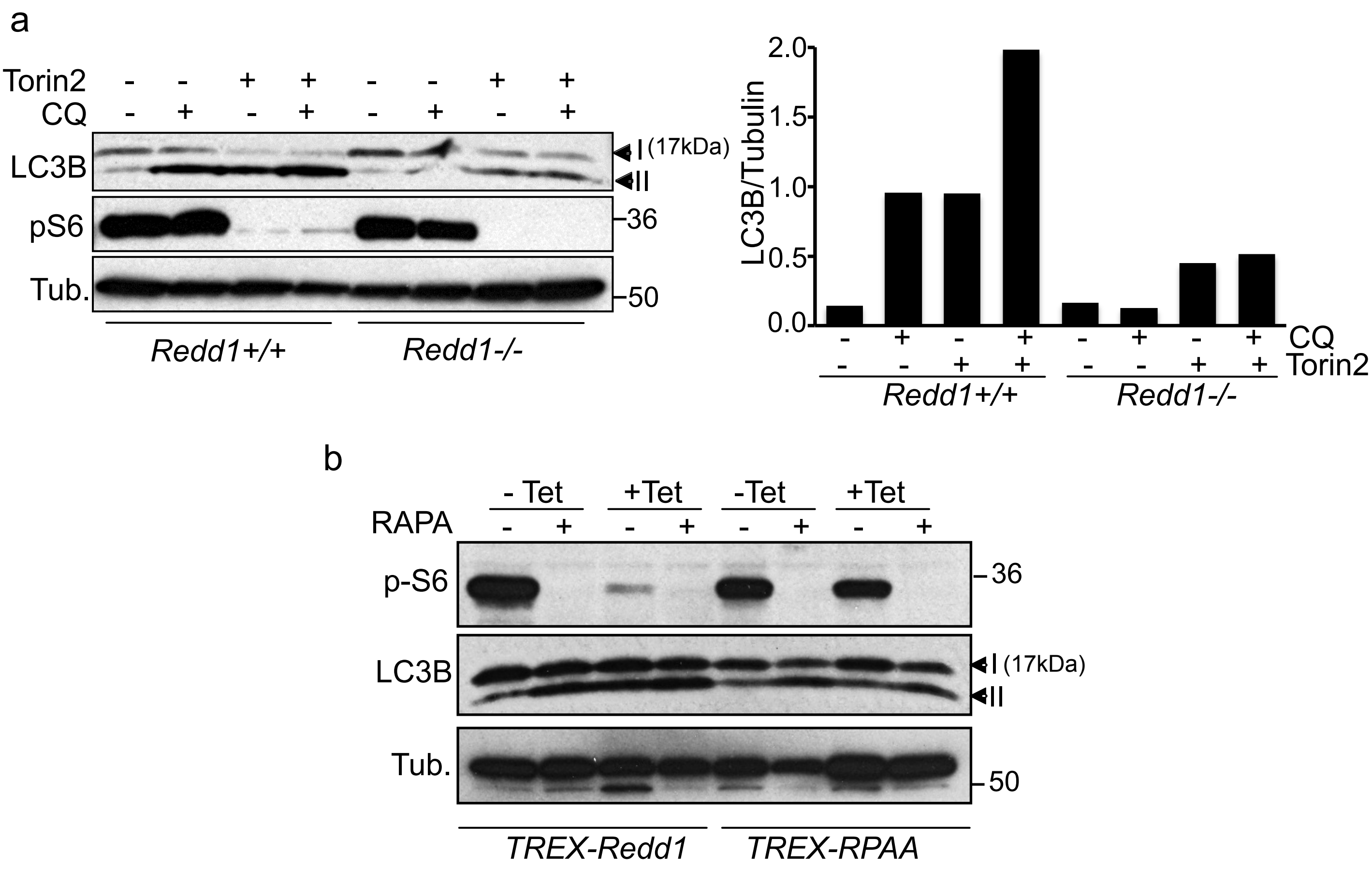


**Supplementary Figure 2. REDD1 regulation of autophagy is largely mTORC1 independent.** (A). Torin2 (25nM, 4h) does not rescue defective autophagic flux in *Redd1*$^{-/-}$ cells, as assessed by LC3B processing in the absence or presence of CQ (30μM, 4h). Activity of mTORC1 was assessed via phosphorylated ribosomal protein S6 (p-S6, S235/S236). β-Tubulin (Tub.) serves as a loading control. This experiment provides additional supportive data to Fig. 2b. Densitometry analysis of LC3B compared to loading control is shown at right. (B). Western blot analysis of endogenous p-S6 and LC3B corresponding to Figure 2d.

Supplementary Figure 3 - Ellisen et al.

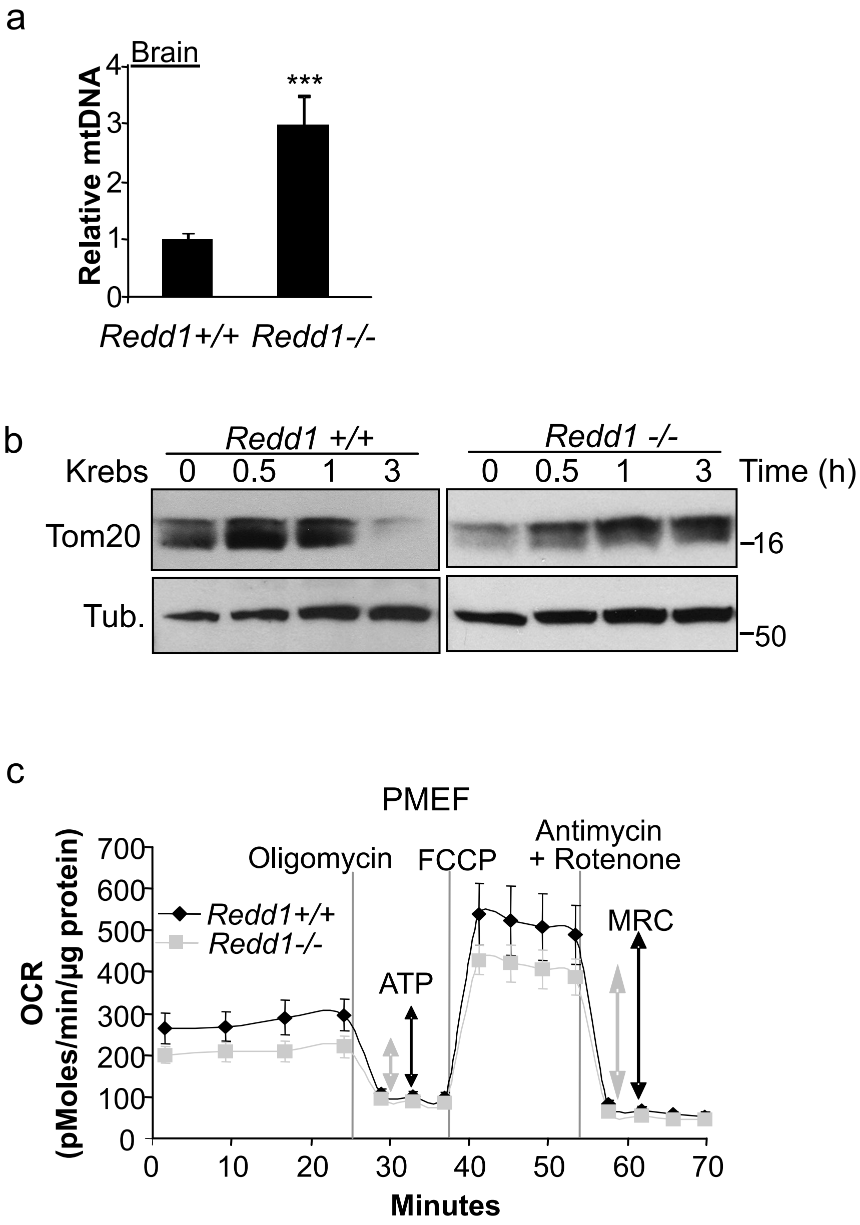


**Supplementary Figure 3. Abnormalities in mitochondrial number and function in the absence of REDD1.** (A). Increased mitochondrial number in brain of *Redd1*$^{-/-}$ mice, evidenced by increased mitochondrial to nuclear (M/N) DNA ratio (COXII/β-globlin) as measured by QRT-PCR. Error bars show SD of three measurements. (B). Failed mitochondrial clearance in *Redd1*$^{-/-}$ cells, evidenced by mitochondrial protein TOM20 levels upon starvation (Krebs solution) in wild-type and *Redd1*$^{-/-}$ MEFs. (C). Decreased basal and maximal O2 consumption rate in primary *Redd1*$^{-/-}$ MEFs. Respiration was assessed in the presence of mitochondrial inhibitors oligomycin (0.25µM), uncoupler FFCP (5µM), antimycin A (1µM) and rotenone (1µM). This experiment shows additional supportive data to Fig. 3f. Error bars show SD of three measurements. ***P < 0.001 by paired t test. (A, C)

Supplementary Figure 4 - Ellisen et al.

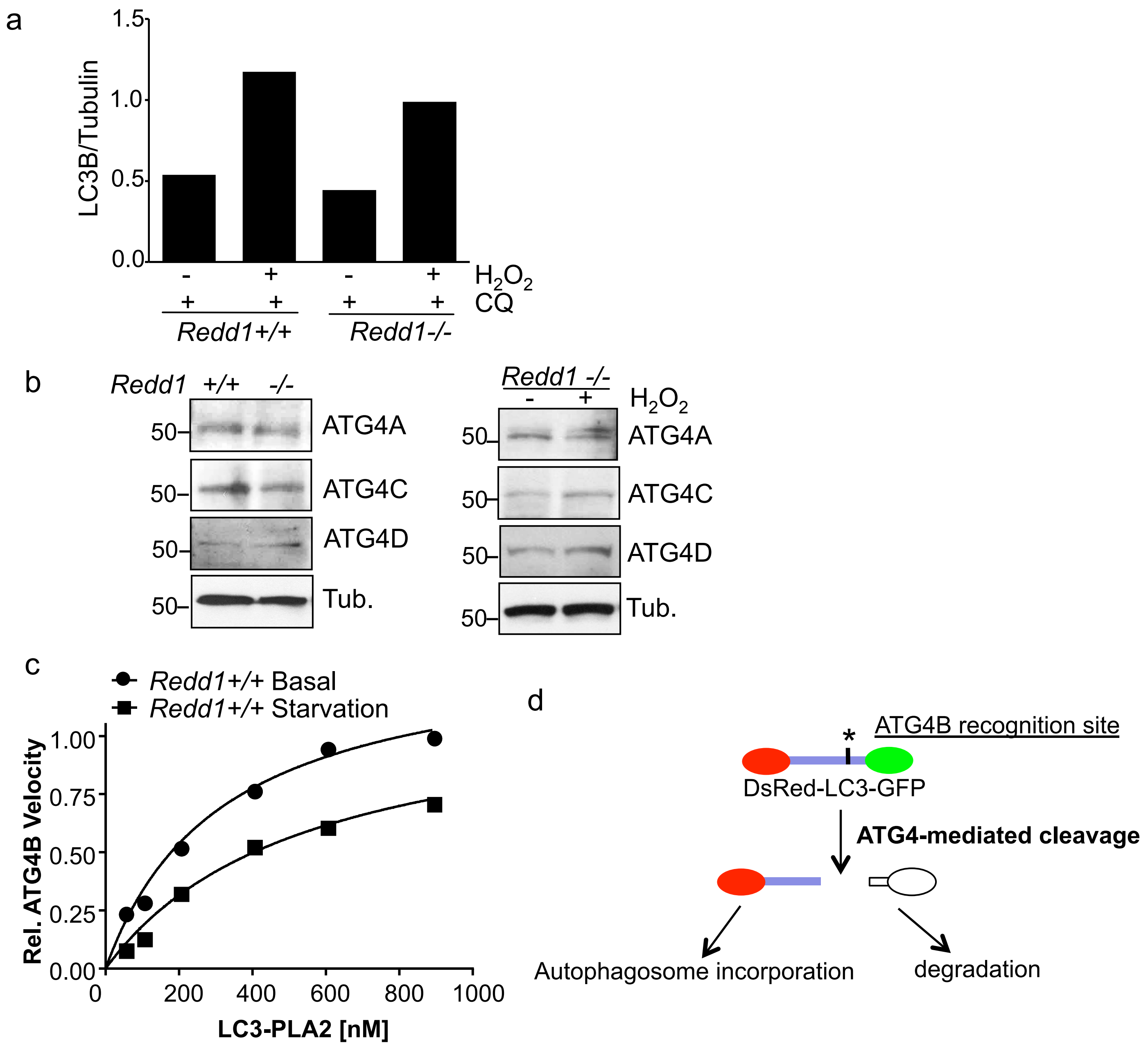


**Supplementary Figure 4. ATG4 levels and suppression of ATG4B activity under starvation.** (A) Densitometry quantitation for Figure 4f. (B) Western blot analysis showing similar levels of ATG4 family members in wild-type and *Redd1*$^{-/-}$ MEFs at basline and following $H_2O_2$ treatment (3mM, 6h). (C) ATG4B activity is suppressed in wild-type cells cultured under starvation (0.1%, FBS, 24h) as compared to cells cultured under basal condition (10% FBS). Cellular extracts were incubated with LC3-$PLA_2$ substrate at indicated concentrations and determination of kinetic parameters for Vmax by nonlinear regression of fitted curves as described in methods. (D) Schematic representation of LC3 processing assaying using double fluorescent tagged DsRed-LC3-GFP. ATG4B cleaves unprocessed LC3 to generate a DsRed-tagged N-terminal fragment and a C-terminal fragment subject to rapid degradation. Thus, the basal GFP/RFP ratio measured by flow cytometry in cells expressing this construct is inversely proportional to ATG4B enzymatic activity.

a

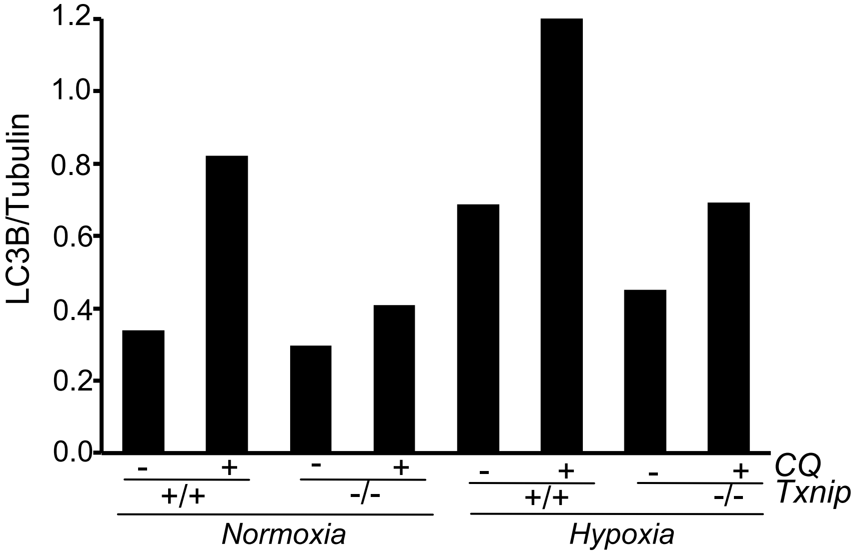


**Supplementary Figure 5. Defective autophagy in *Txnip*$^{-/-}$ cells.**
Densitometry quantitation of LC3B compared to loading control for Figure 5h.

Supplementary Figure 6 - Ellisen et al.

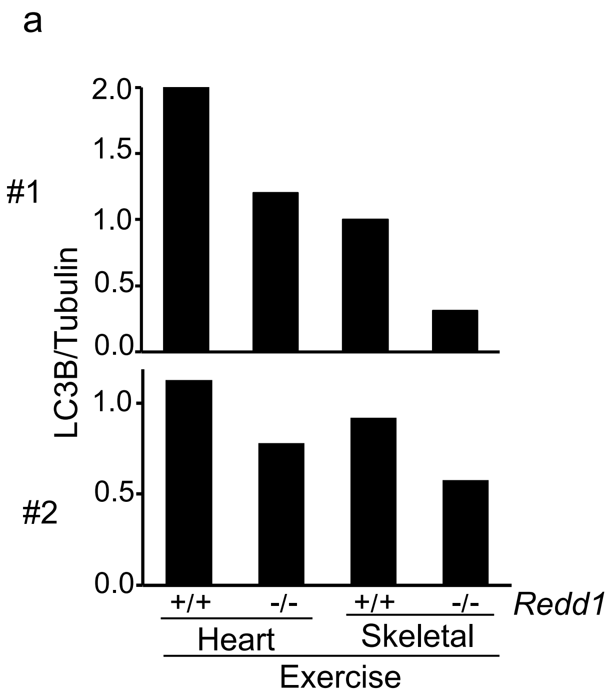


**Supplementary Figure 6. Defective autophagy in *Redd1*$^{-/-}$ tissue.**
Densitometry quantitation of LC3B compared to loading control for Figure 6f.

# Supplementary Figure 7 - Uncropped blots

Fig 1a

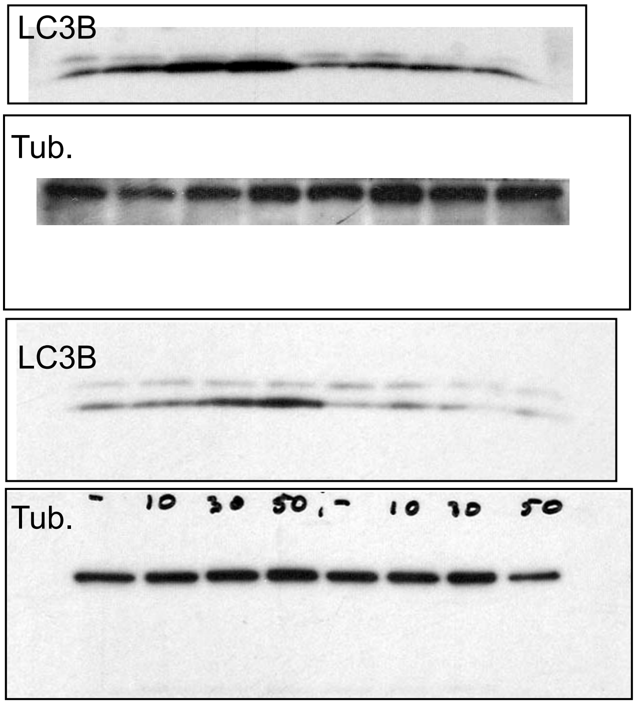


Fig 1c

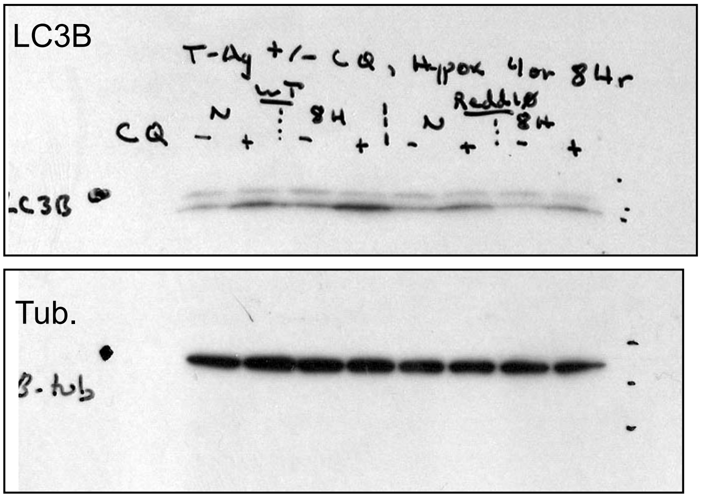


Fig 1e

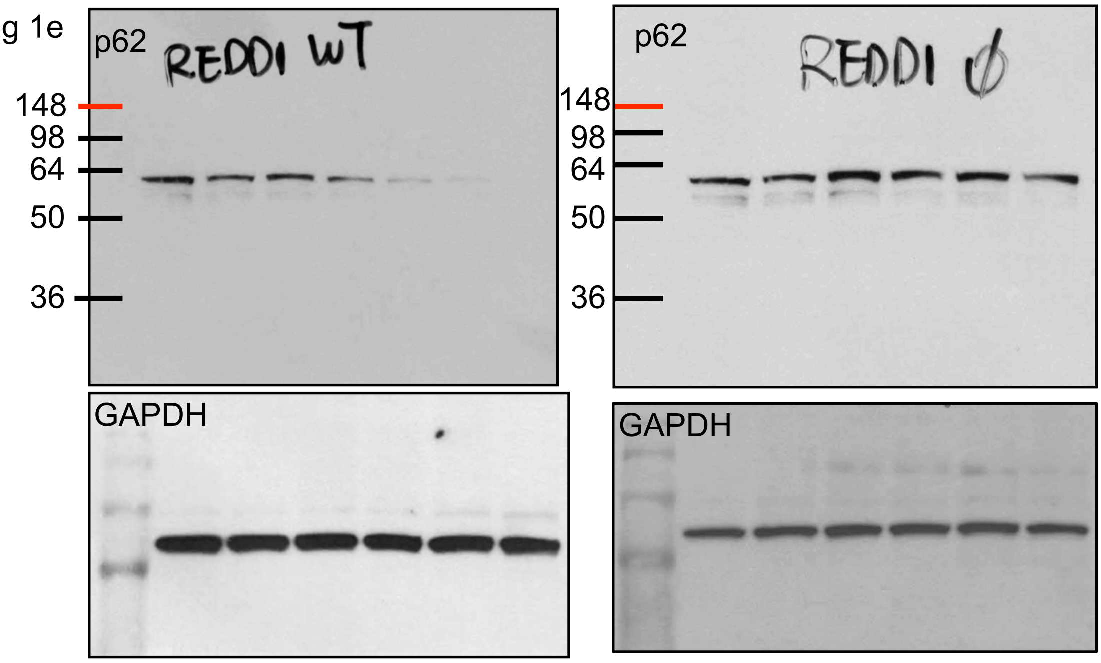


Fig 1f

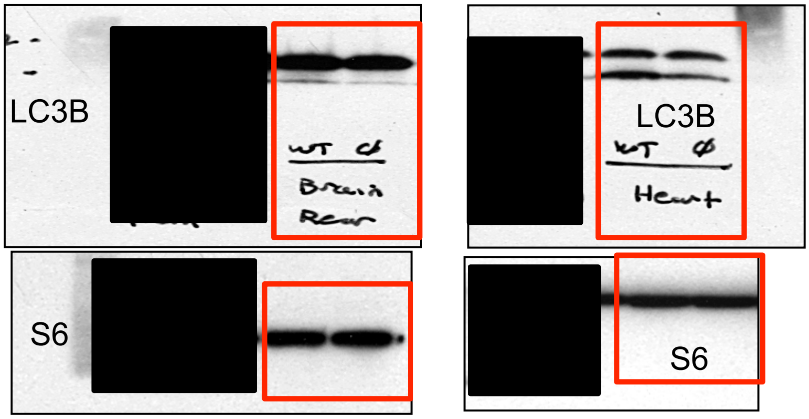

Supplementary Figure 7 - Uncropped blots

Fig 1f

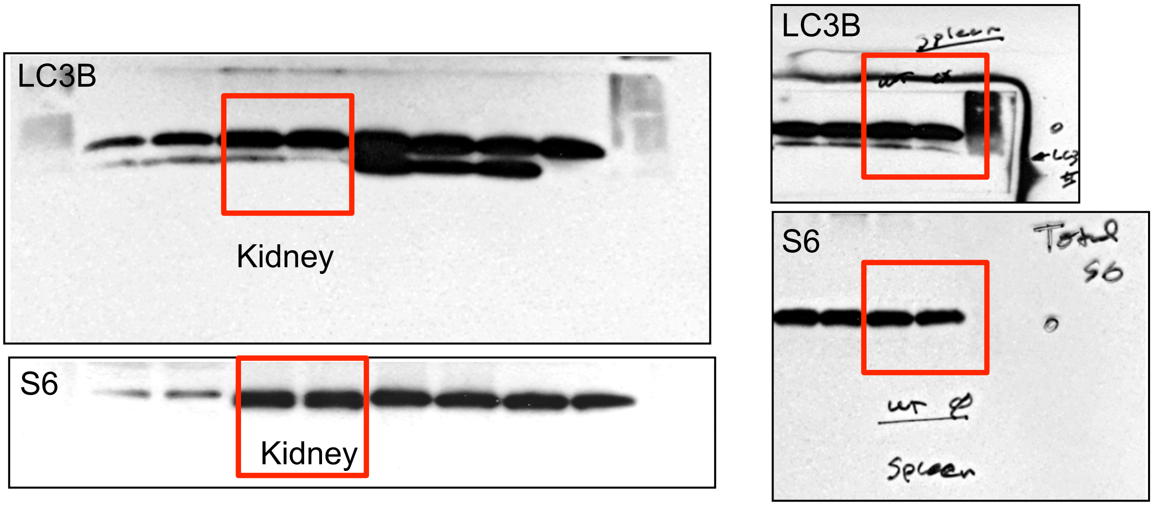


Fig 3a

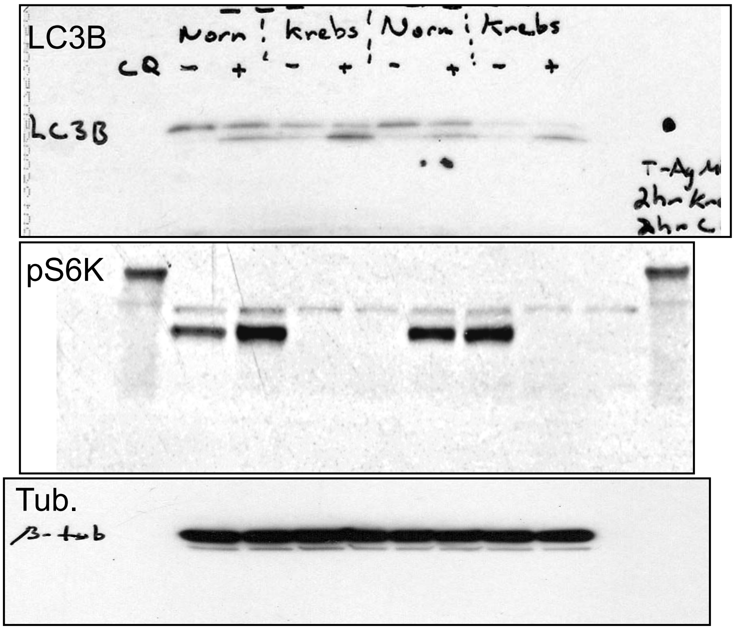


Fig 3b

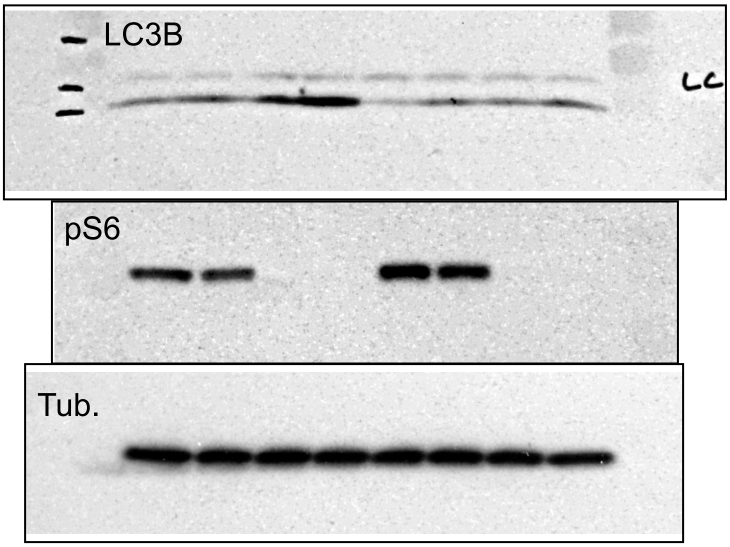

Supplementary Figure 7 - Uncropped blots

Fig 4f

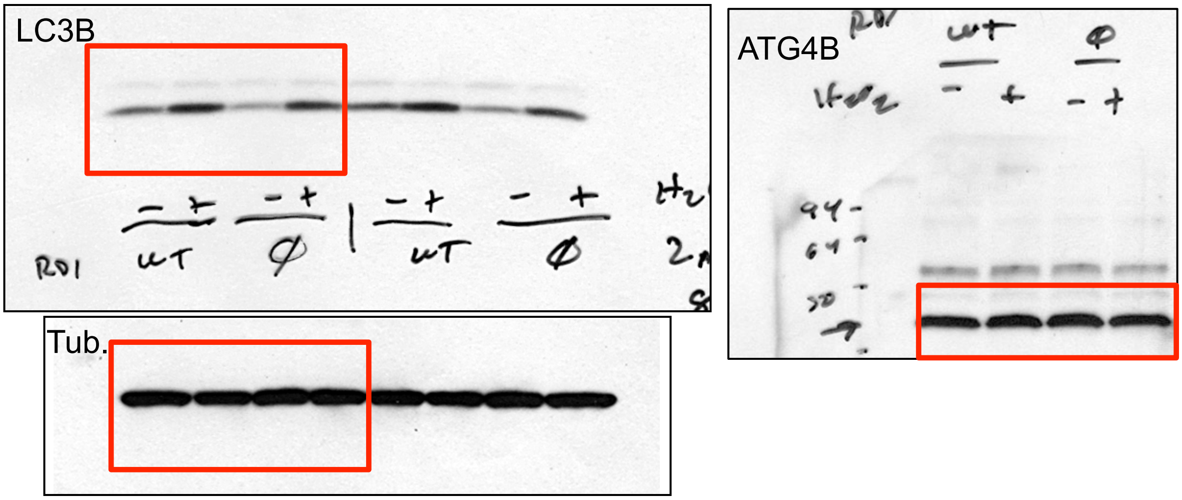


Fig 5h

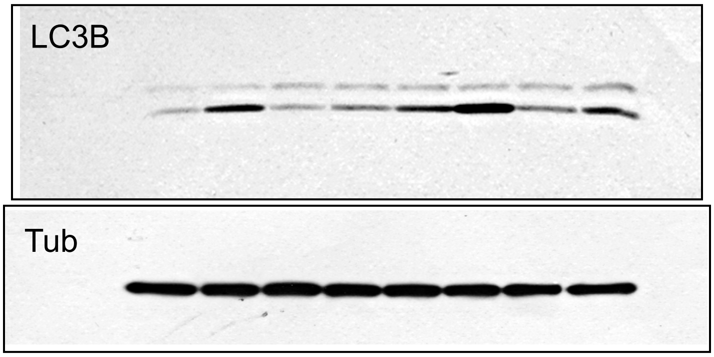


Fig 5j

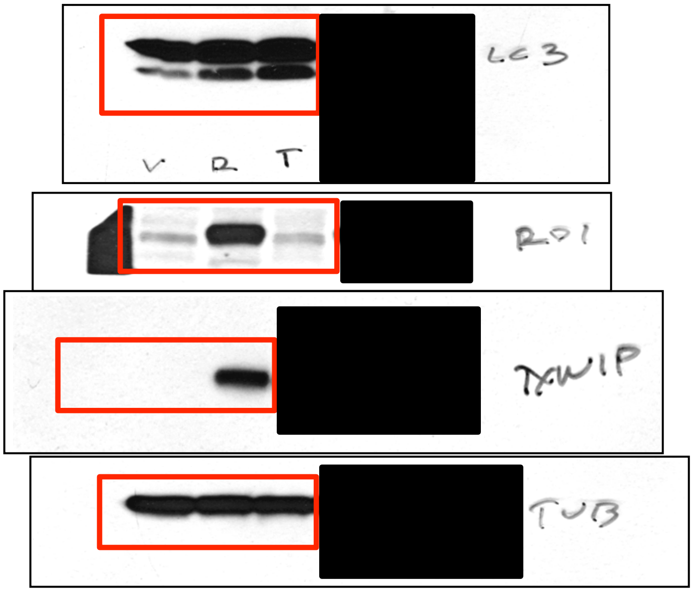

# Supplementary Figure 7 - Uncropped blots

Fig 6a

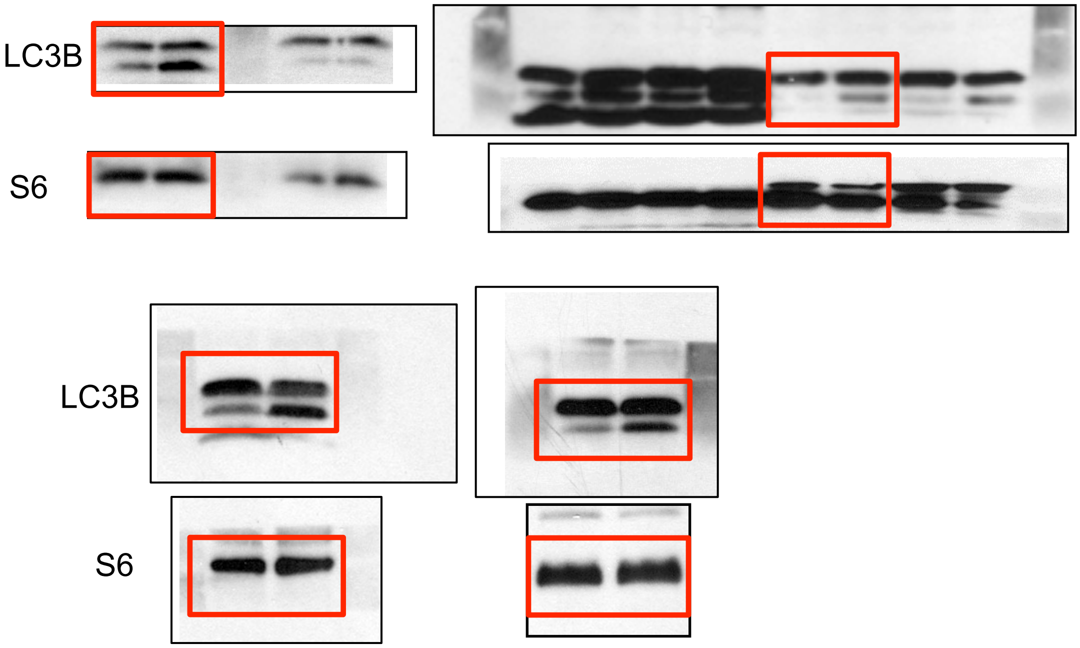


Fig 6f

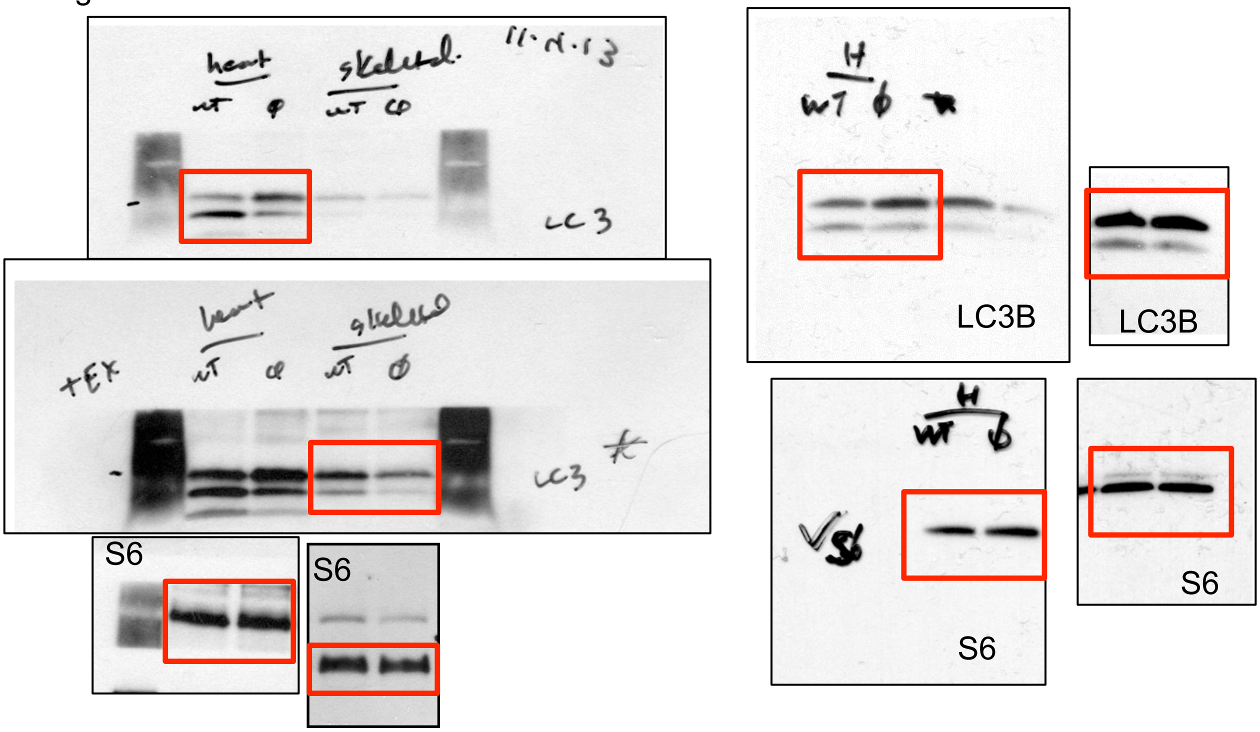

Supplementary Figure 7 - Uncropped blots

Fig S1d

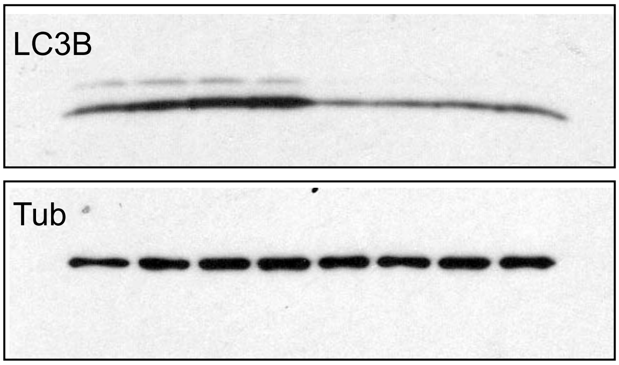


Fig S2a

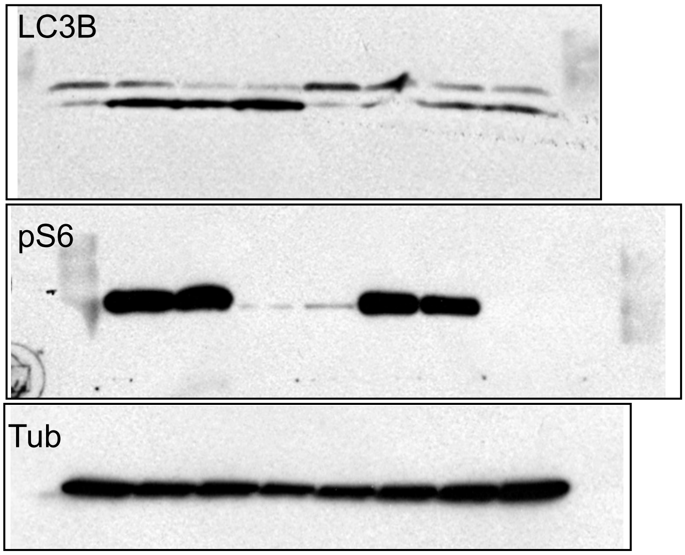


Fig S3b

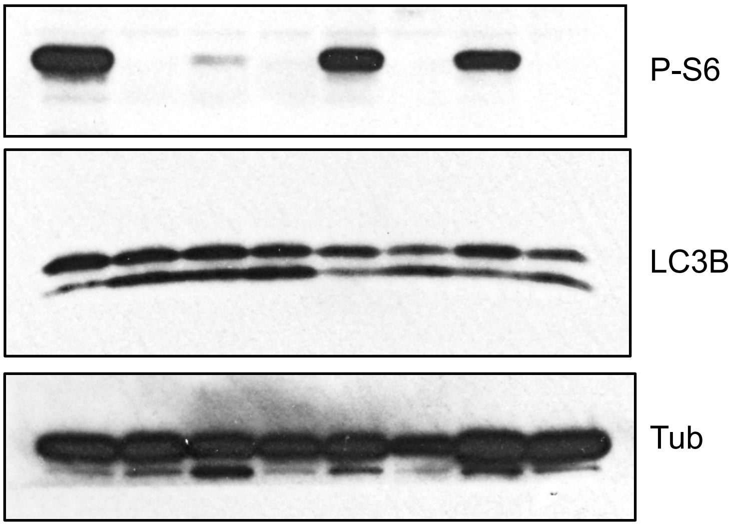

# Supplementary Figure 7 - Uncropped blots

Fig S3b

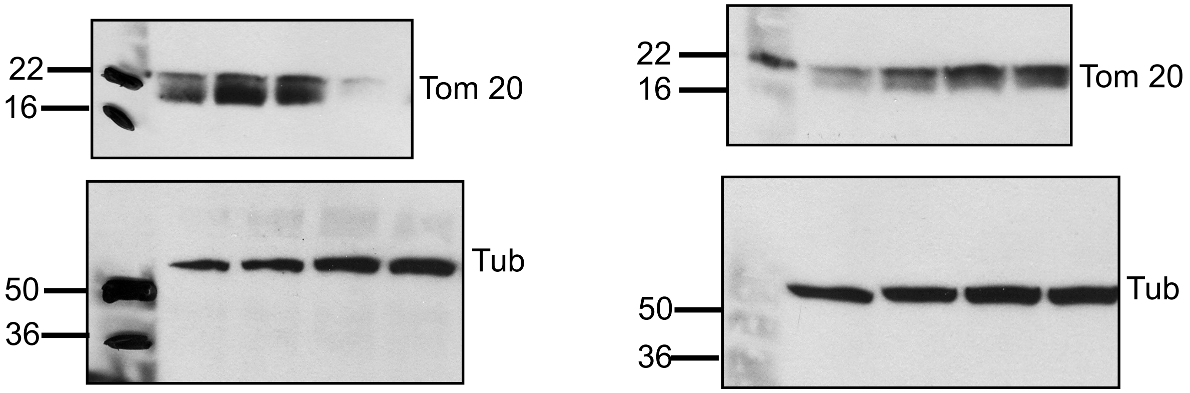


Fig S4b

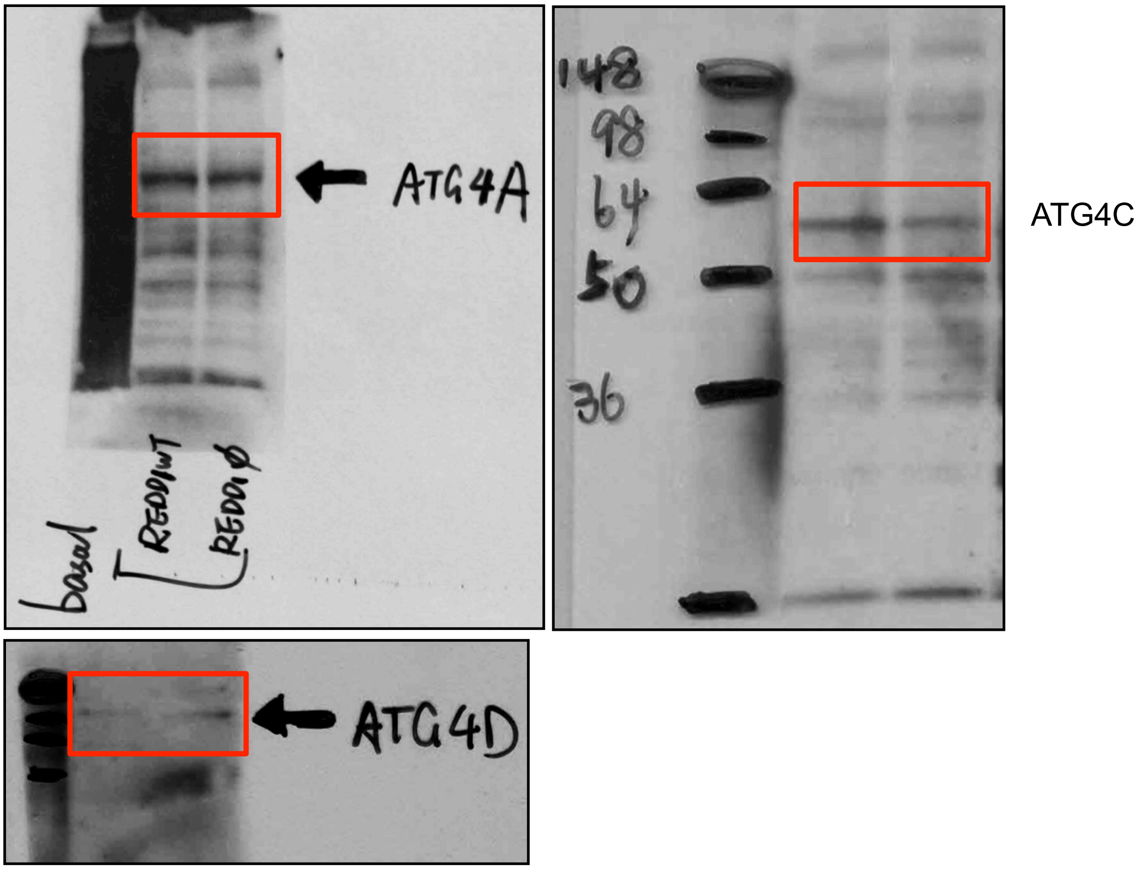

# Supplementary Figure 7 - Uncropped blots

Fig S4b

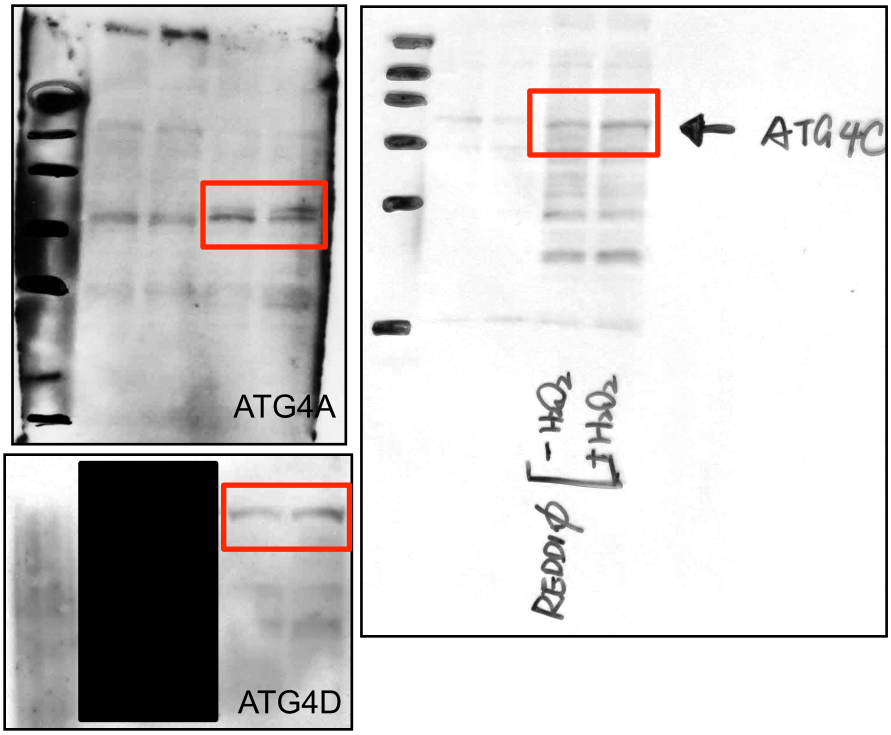

Supplement: Supplementary Information — Supplementary Figures 1-7 [file ncomms8014-s1.pdf]
